# Genetic variations in relation to bleeding and pharmacodynamics of dabigatran in Chinese patients with nonvalvular atrial fibrillation: A nationwide multicentre prospective cohort study

**DOI:** 10.1002/ctm2.1104

**Published:** 2022-12-01

**Authors:** Qian Xiang, Qiufen Xie, Zhiyan Liu, Guangyan Mu, Hanxu Zhang, Shuang Zhou, Zhe Wang, Zining Wang, Yatong Zhang, Zinan Zhao, Dongdong Yuan, Liping Guo, Na Wang, Jing Xiang, Hongtao Song, Jianjun Sun, Jie Jiang, Yimin Cui

**Affiliations:** ^1^ Department of Pharmacy Peking University First Hospital Beijing China; ^2^ School of Pharmaceutical Sciences Peking University Health Science Center Beijing China; ^3^ Department of Pharmacy Beijing Hospital Beijing China; ^4^ Department of Pharmacy Zhengzhou Seventh People's Hospital Zhengzhou China; ^5^ Department of Pharmacy The Second Affiliated Hospital of Chongqing Medical University Chongqing China; ^6^ Department of Pharmacy 900 Hospital of the Joint Logistics Team Fuzhou China; ^7^ Department of Pharmacy The Affiliated Hospital of Inner Mongolia Medical University Huhehaote China; ^8^ Department of Cardiology Peking University First Hospital Beijing China; ^9^ Institute of Clinical Pharmacology Peking University Beijing China

**Keywords:** atrial fibrillation, bleeding, dabigatran, genome‐wide association analysis, pharmacodynamics, whole‐exome sequencing

## Abstract

**Introduction:**

To identify the potential factors responsible for the individual variability of dabigatran, we investigated the genetic variations associated with clinical outcomes and pharmacodynamics (PD) in Chinese patients with nonvalvular atrial fibrillation (NVAF).

**Materials and methods:**

Chinese patients with NVAF taking dabigatran etexilate with therapeutic doses were enrolled. The primary (bleeding events) and secondary (thromboembolic and major adverse cardiac events) outcomes for a 2‐year follow‐up were evaluated. Peak and trough PD parameters (anti‐FIIa activity, activated partial thromboplastin time and prothrombin time) were detected. Whole‐exome sequencing, genome‐wide sequencing and candidate gene association analyses were performed.

**Results:**

There were 170 patients with NVAF treated with dabigatran (110 mg twice daily) who were finally included. Two single‐nucleotide polymorphisms (SNPs) were significantly related with bleeding, which include *UBASH3B* rs2276408 (odds ratio [OR] = 8.79, 95% confidence interval [CI]: 2.99–25.83, *p* = 7.77 × 10^−5^ at sixth month visit) and *FBN2* rs3805625 (OR = 8.29, 95% CI: 2.87–23.89, *p* = 9.08 × 10^−5^ at 12th month visit), as well as with increased trends at other visits (*p* < .05). Furthermore, minor allele carriers of 16 new SNPs increased PD levels, and those of one new SNP decreased PD values (*p* < 1.0 × 10^−5^). Lastly, 33 new SNPs were found to be associated with bleeding and PD among 14 candidate genes. Unfortunately, the low number of secondary outcomes precluded further association analyses.

**Conclusions:**

Genetic variations indeed affected bleeding and PD in Chinese patients with NVAF treated with dabigatran. The functions of these suggestive genes and SNPs might further be explored and verified in more in vivo and in vitro investigations.

## Graphical abstract

- Genetic variations indeed affected outcomes of dabigatran in Chinese NVAF patients.
- Minor allele carriers of *UBASH3B* rs2276408 and *FBN2* rs3805625 increased bleeding risk.
- Seventeen identified SNP polymorphisms were associated with pharmacodynamics.
- Fourteen reported candidate genes were associated with bleeding and pharmacodynamics.

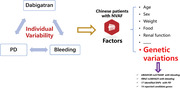

## 1 INTRODUCTION

Atrial fibrillation (AF) is becoming more prevalent globally in persistent arrhythmia of adults and is worthy of attention with its serious risk of stroke and all‐cause mortality.^1^, ^2^ Direct‐acting oral anticoagulant, dabigatran, inhibits factor IIa activity directly and has been recommended as one of the preferable therapies for individuals with nonvalvular AF (NVAF) to prevent stroke or systemic embolism (SE) in the global guidelines.^3^, ^4^ With generic drug development and drug price reductions in China, dabigatran is becoming more widely prescribed for patients with NVAF in the recent years.^5^ Whereas, the controversial issues between the fixed‐dose regimens and its variations on pharmacokinetics (PK), pharmacodynamics (PD) and clinical outcomes have always been discussed. Previous studies in healthy participants demonstrated interindividual coefficient of variations (CVs) in PK and PD being 8.4%–46.0% and usually below 20%, respectively.^6^, ^7^ Besides, in the real world of patients with AF administered with recommended doses of dabigatran, the trough and peak plasma levels of interpatient CVs were 63.8% and 50.9%, respectively, and intrapatient CVs were 32.9% and 39.5%, respectively.^8^ Furthermore, Asian patients receiving dabigatran 110 or 150 mg twice daily had a lower incidence of stroke and SE, haemorrhagic stroke and all‐cause mortality compared with non‐Asian patients.^9^

So far, except for the meaningful factors responsible for individual variability in dabigatran, including age, sex, weight, food and renal function,^10^, ^11^, ^12^ we could not ignore the pharmacogenomic influence on this issue.^13^, ^14^, ^15^, ^16^, ^17^ Considering mainly the metabolism characteristics, previous studies were mainly devoted to verify the impact of genetic variations in the *ABCB1* and *CES1* polymorphism on dabigatran in different populations. *ABCB1* gene encodes P‐glycoprotein (P‐gp) whose substrates, including dabigatran, and *CES1* gene encode carboxylesterase 1, which primarily activates and hydrolyzes the prodrug (dabigatran etexilate) in hepatocytes.^13^ However, whether in healthy participants or in AF populations, the significant impact of these two genes on the outcomes were not totally consistent (Table S1). Besides, there were only two researches that involved other genes except *ABCB1* and *CES1* before 2022, and no significant effect on PD and clinical outcomes was found.^18^, ^19^

Considering the different distribution of genes and analytical association methods,^20^, ^21^ we previously performed a pharmacogenomic study in 118 healthy Chinese volunteers to investigate the meaningful single‐nucleotide polymorphisms (SNPs) associated with dabigatran metabolism.^22^ For further exploring the related genetic variations in patients, we planned to carry out a nationwide multicentre prospective cohort study in Chinese patients with NVAF by whole‐exome sequencing and genome‐wide association (GWA) analyses. We hope to seek for more significant markers on dabigatran efficacy and safety and improve the individualized anticoagulant therapy regimen in AF.

## 2 MATERIALS AND METHODS

### 2.1 Study design

This prospective research was conducted in a nationwide multicentre of six hospitals in China. The protocol registered in ClinicalTrial.org (NCT03161496) was approved by the ethics committees of all the hospitals. After introduction of the detailed information about the research, written informed consent was obtained from each patient before enrolment.

Adult patients with NVAF who had planned to take or had been taking dabigatran etexilate (brand name: Pradaxa) were recruited from September 2017 to May 2020. To ensure a steady drug concentration before blood sample collection, patients who had planned to take drug were required not to take it within 1 month before the study. On the other hand, patients who had been taking it were required to administer it continuously for more than 1 week prior to the beginning of the study. The exclusion criteria were as follows: (i) an immunodeficiency disease history, including positive human immunodeficiency virus (HIV) indices; (ii) co‐medications including CYP3A4 strong inhibitors and P‐gp inhibitors, and CYP3A4 strong inducers and P‐gp inducers (examples of detailed drugs showed in ClinicalTrial.org) within 14 days before dabigatran treatment; (iii) severe abnormal liver function (Child–Pugh B/C and liver cirrhosis) or renal function (estimated glomerular filtration rate [eGFR] <30 ml/min*1.73 m^2^); and (iv) any dabigatran contraindications, including hypersensitivity, active bleeding, history of intracranial and gastrointestinal (GI) haemorrhage within previous 6 months, and any major operations in the past 30 days.

After enrolment, each patient was required to take dabigatran regularly at therapeutic doses (110 or 150 mg twice daily) prescribed by his/her physician. They were also required to provide blood samples for the detection of genotypes and PD parameters after reaching steady drug concentrations (regularly taking dabigatran daily at least 72 h), and were followed up regularly for 2 years. Moreover, other related information was obtained from medical records, including demographics, clinical examinations indices, co‐medications and comorbidities at the time of enrolment. CHA_2_DS_2_‐VASc and HAS‐BLED scoring systems were used to evaluate the risks of thromboembolism and haemorrhage, respectively. The formula of eGFR determined was the CKD‐EPI (Chronic Kidney Disease Epidemiology Collaboration) equation.^23^

### 2.2 Pharmacodynamic evaluation

PD outcomes were mainly evaluated via trough and peak prothrombin time (PT), activated partial thromboplastin time (APTT) and anti‐FIIa activity. After reaching a steady state of dabigatran and without any dose adjustments, blood samples (2.7 ml) were taken in sodium citrate (3.2% v/v) tubes at time points >10 h after previous dose for detecting trough PD and 2 h after dosing for peak PD, respectively. Within 1 h after collection, blood samples were centrifuged for 15 min in 2500 × *g* at room temperature. The plasma samples were then stored at −80°C at each centre and all the samples were transferred to Peking University First Hospital for PD tests within 6 months. Through automated multiparameter haemostasis analyzer (Sysmex CS‐2100i), validated Coagulation or Chromogenic Method Kits were used to measure PT, APTT and anti‐FIIa activity. The detailed and validated methods of determining these parameters have been published in our previous studies.^22^, ^24^

### 2.3 Genotyping

Blood samples (6 ml) for genotype tests were taken in EDTA‐K_2_ tubes when collecting trough PD samples. Subsequently, the whole‐blood samples from all centres were stored at less than −60°C till genotype tests were conducted in the same institution (CapitalBio Technology Co., Ltd, Beijing, China). After genomic DNA preparation and quality assessment, whole‐exome sequencing was used for genotyping, which has also been published in our previous study.^22^ Agilent SureSelect Human All Exon V6 Kit (Agilent Technologies Inc., USA) was used to create the whole‐exome library, and an Illumina NovaSeq 6000 sequencer (Illumina, USA) was used for paired‐end sequencing (2 × 150 bp). With 200‐bp extensions on either end, 283 350 SNPs found in exons underwent variant filtering and prediction. Missing rate more than 10%, minor allele frequency (MAF) less than 5% and Hardy–Weinberg equilibrium *p*‐value <10^−6^ were used as exclusion criteria of SNPs. Principal component analysis (PCA) was used in PLINK 1.09 to perform a stratified population evaluation and eliminate outlier samples (nine samples). Eventually, 75 630 SNPs were retained for correlation analysis of PD and clinical outcomes.

### 2.4 Follow‐up and clinical outcomes

After enrolment, all patients were followed up through telephone call or office appointment at 1, 6, 12 and 24 months; follow‐up was completed once they discontinued dabigatran. At every visit, the clinical outcomes, treatment compliance and co‐medications were required to be collected in detail with the medical records. The primary outcome was any bleeding event defined by the criterion of Bleeding Academic Research Consortium (BARC).^25^ Major bleeding was determined as BARC types 3, 4 and 5 events, and BARC types 1 and 2 events were considered as minor bleeding. The secondary outcome was the incidence of thromboembolic events (TEs), including myocardial infarction, stroke or transient ischaemic attack, SE, pulmonary embolism, and all‐cause mortality, and major adverse cardiac events (MACEs) comprising cardiac death, myocardial infarction, stroke, stent thrombosis and repeated revascularization. Two independent doctors who were blinded to the findings of genotyping and PD tests assessed every event.

### 2.5 Statistical analyses

Genome‐wide analyses for PD parameters and clinical outcomes were performed using linear regression and logistic regression methods, respectively, in PLINK1.09, assuming an additive genetic model.^26^ PCA was conducted to correct possible population stratification and exclude any outliers.^27^ Covariates of PD indices were also further adjusted for sex, creatinine, catheter ablation and mean platelet volume. Logistic regression models included sex and creatinine as independent variables. These clinical variables were selected, as they were associated with PD parameters and the clinical outcomes in univariate analyses and multivariate analyses. The genome‐wide *p*‐value significant threshold was set at .05/75 630 = 6.61 × 10^−7^ using a Bonferroni correction for successful replication. In addition, candidate gene association analyses were also used to verify meaningful genes related with PD and clinical outcomes of dabigatran. From prior pharmacogenomic researches (Table S1) and reviews,^13^, ^17^ a total of 25 candidate genes were chosen, including *ABCB1*, *ABCC2*, *ABCG2*, *CES1*, *CES1P2*, *CYP1A2*, *CYP2A6*, *CYP2B6*, *CYP2C19*, *CYP2C8*, *CYP2C9*, *CYP2D6*, *CYP2J2*, *CYP3A4*, *CYP3A5*, *CYP4F2*, *FRAS1*, *SLC22A1*, *SLC4A4*, *SLCO1B1*, *SULT1A1*, *UGT1A1*, *UGT1A9*, *UGT2B7* and *UGT2B15*. The detailed methods about genome‐wide and candidate gene association analyses were similar to those used in our GWA study on healthy participants with dabigatran.^22^ Regional plots were created in LocusZoom,^28^ and additional graphics were generated using R. Except otherwise specified, continuous variables were presented as mean ± standard deviation, and a two‐sided *p*‐value of less than .05 was regarded as statistically significant.

## 3 RESULTS

### 3.1 Patient characteristics and outcomes

Overall, 170 patients with NVAF were included in our final genetic analysis. Basic characteristics and outcomes are showed in Table 1. The median age was 72 years, and 133 (78.2%) patients were aged 65 years or above. The median eGFR level was 73.6 ml/min/1.73 m^2^, and 167 (98.8%) of the levels were greater than or equal to 30 ml/min/1.73 m^2^. The dosage regimen of dabigatran was 110 mg twice daily for all. The median and maximum scores of CHA_2_DS_2_‐VASc were 2 and 8, and 155 (91.2%) scores were greater than or equal to 2. The median and maximum scores of HAS‐BLED were 4 and 5, and 64 (37.6%) of the scores were greater than or equal to 3. The median follow‐up duration was 12 months, and 150 (88.8%) visits were in greater than or equal to 6 months. A total of 151 (88.8%) and 155 (91.2%) data points were obtained for trough and peak PD tests, respectively, and 156 points for peak anti‐FIIa activity. The trough levels of all three PD parameters were significantly lower than each peak level (all *p* < .001). Bleeding events during follow‐up had a higher incidence compared with TEs and MACEs. The detailed clinical outcomes from each follow‐up visit are displayed in Table S2. There were only two major bleedings reported at visit on the 12th month, which were haematuria intervened by surgery and intracranial haemorrhage. Most of the remaining minor bleedings contained gingival, subcutaneous, nasal, pharyngeal, conjunctival, GI bleeding, microscopic haematuria and haematuria without intervention. Some patients experienced more than two types of bleeding. For TEs and MACEs, the events were focused on ischaemic strokes, haemorrhagic strokes, myocardial infarction, SE and repeated revascularization. Moreover, no patients suffered more than two types of the above events. Considering the low incidence of TEs and MACEs as well as that of major bleedings, we only performed GWA analysis for primary outcomes of all cumulative bleeding events.

**TABLE 1:** Basic characteristics and outcomes of patients included in genome‐wide association analysis

**Variables**	**Results**
**Baseline characteristics**	
Total patients	170
Regimen of dabigatran	110 mg (*n* = 170), twice daily
Female, *n* (%)	67 (39.4)
Age (years)	72.0 (65.8, 79.0)
Bodyweight (kg)^a^	70.7 ± 12.51
BMI (kg/m^2^)^a^	25.4 ± 3.69
CREA (μmol/L)^a^	83.8 ± 19.51
eGFR (ml/min/1.73 m^2^)^a^	73.6 (58.6, 88.6)
ALT (IU/L)^a^	19.0 (14.0, 27.4)
AST (IU/L)^a^	20.0 (16.0, 25.0)
HGB (g/L)^a^	136.0 (121.5, 147.0)
PLT (10^9^/L)^a^	195.0 (162.0, 239.0)
MPV (fl)^a^	9.4 (8.4, 10.9)
EF (%)^a^	63.9 (58.8, 70.6)
CHA_2_DS_2_‐VASc score	2.0 (1.75, 3.0)
HAS‐BLED score	4.0 (3.0, 5.0)

Comorbidities, *n* (%)	
Hypertension	121 (71.2)
Diabetes mellitus	58 (34.1)
Stroke or TIA	56 (32.9)
Coronary heart disease	76 (44.7)
Myocardial infarction	7 (4.1)
Heart failure	27 (15.9)
PCI	14 (8.2)
Catheter ablation^a^	39 (34.8)

Co‐medications, *n* (%)	
ACEIs or ARBs^a^	73 (43.5)
Beta‐blockers^a^	95 (56.9)
Calcium channel blockers^a^	64 (38.3)
Statins^a^	103 (61.7)
Antiplatelet drugs^a^	19 (11.3)
Nitrate esters^a^	14 (12.1)
Proton‐pump inhibitors^a^	33 (19.8)

Pharmacodynamic parameters		
	Trough levels	Peak levels
**Patients with tests, *n* (%)**	151	155
Anti‐IIa activity (ng/ml)	68.16 ± 60.34	150.62 ± 128.00^b^
APTT (s)	39.55 ± 9.90	48.29 ± 13.73
PT (s)	13.72 ± 6.11	14.09 ± 3.62

Events, *n* (%)			
	Bleeding	TE	MACE
0–1 month (*n* = 169)	7 (4.1)	2 (1.2)	0 (0.0)
2–6 months (*n* = 149)	11 (7.4)	2 (1.3)	2 (1.3)
7–12 months (*n* = 122)	12 (9.8)	3 (2.5)	4 (3.3)
13–24 months (*n* = 51)	3 (5.9)	2 (3.9)	2 (3.9)

*Note*: For the continuous variables in baseline characteristics, data in normal distribution are shown as ‘mean ± SD’, and in skewed distribution are shown as ‘median (25, 75 percentiles)’.

Abbreviations: ACEI, angiotensin‐converting enzyme inhibitor; ALT, alanine aminotransferase; APTT, activated partial thromboplastin time; ARB, angiotensin receptor blockers; AST, aspartate aminotransferase; BMI, body mass index; CREA, creatinine; EF, ejection fraction; eGFR, estimate glomerular filtrate rate; HGB, haemoglobin; MACE, major adverse cardiac event; MPV, mean platelet volume; PCI, percutaneous coronary intervention; PLT, platelet; PT, prothrombin time; TE, thromboembolic event; TIA, transient ischaemic attack.

^a^ The number of the variable was the number of observed values, excluding missing values.

^b^ The number of peak anti‐IIa activity tests was 156.

### 3.2 Effects of suggestive genetic variations on bleeding events of dabigatran

**FIGURE 1:**
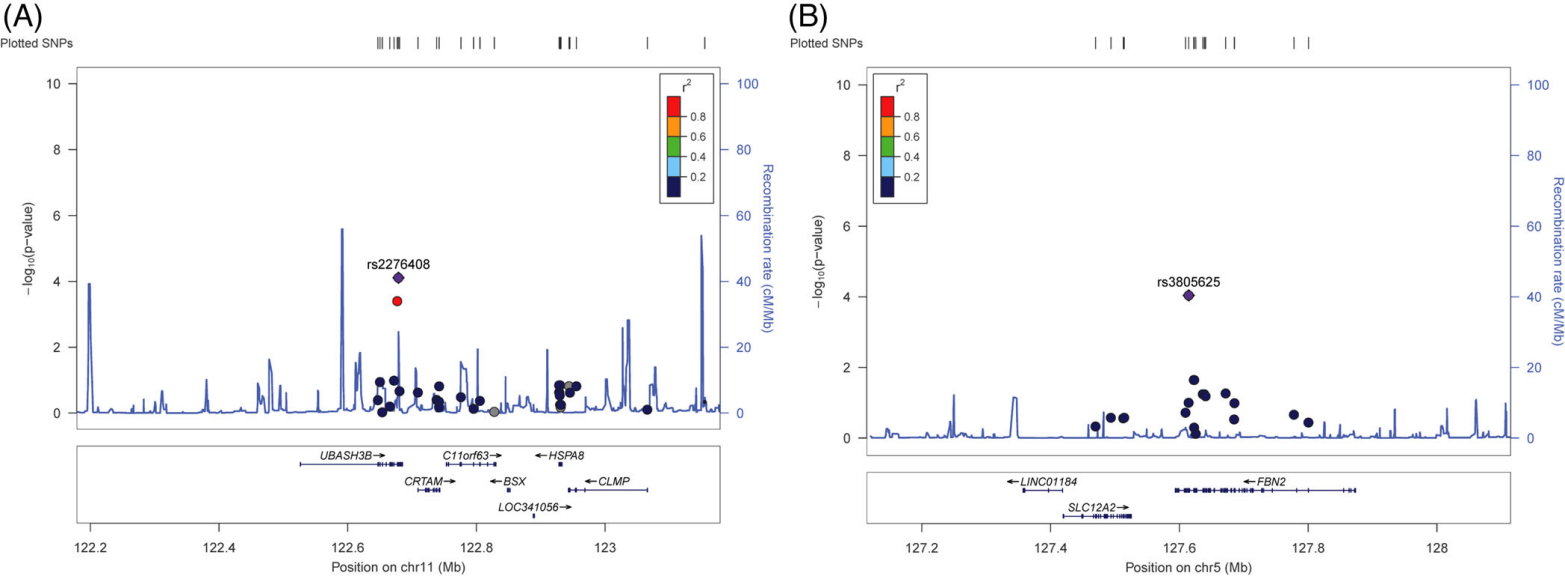
Regional association plots of suggestive single‐nucleotide polymorphisms (SNPs) on bleeding events of patients treated with dabigatran (A) *UBASH3B* SNP rs2276408 (B) *FBN2* SNP rs3805625. Annotation: SNPs are presented as per their physical location and –log_10_ *p*‐values for association. The recombination rate is also shown in centimorgans per megabase (blue line) and the linkage disequilibrium (*r* ^2^) of each SNP with the SNP having the lowest *p*‐value.

**FIGURE 2:**
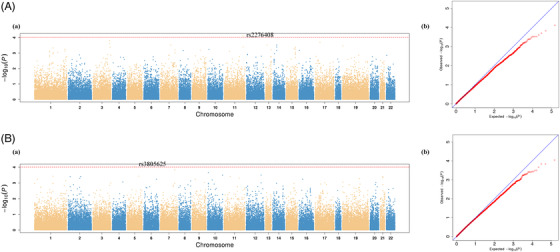
Manhattan and quantile–quantile plots of the association with bleeding events of patients treated with dabigatran. (A) *UBASH3B* SNP rs2276408. (B) *FBN2* SNP rs3805625. Annotation: (a) Manhattan plot; (b) Quantile–quantile plot

In the light of modest sample size and incidence rates of this exploratory genetic study, the genome‐wide *p*‐value significant threshold was adjusted to 1.0 × 10^−4^ for screening suggestive genetic variations on cumulative bleeding events at each follow‐up. Eventually, two SNPs were related to bleeding events, including *UBASH3B* rs2276408 (odds ratio [OR] = 8.79, 95% confidence interval [CI]: 2.99–25.83, *p* = 7.77 × 10^−5^ at the 6th month visit) and *FBN2* rs3805625 (OR = 8.29, 95% CI: 2.87–23.89, *p* = 9.08 × 10^−5^ at the 12th month visit). The detailed information of these genetic variations on bleeding is showed in Table 2. Besides, Figure 1 presents regional association plots within 500 kilobases, and Figure 2 displays Manhattan plots as well as quantile–quantile (Q–Q) plots. Except for the above‐selected visit, the minor allele carriers of both of these two SNPs significantly increased the risk of bleeding events at visits on 6, 12 and 24 months (*p* < .05). At their first month visit, the minor allele carriers of *UBASH3B* rs2276408 also had an increased risk of bleeding (*p* < .05). We also performed analysis of these genetic variations on peak and trough PD (Table S3). It was only *UBASH3B* rs2276408 that is significantly associated with peak anti‐FIIa activity (*p* = .024). The minor allele carriers showed a significantly higher peak anti‐FIIa level, and this same trend was also present in peak APTT, peak PT and trough PT with no significant differences.

**TABLE 2:** Effects of suggestive genetic variations on bleeding events of patients treated with dabigatran

Gene	*UBASH3B*						*FBN2*					
SNP	rs2276408						rs3805625					
CHR	11						5					
Func	Intronic						Intronic					
Bleeding events, *n* (%)	GENO	A1A1	A1A2	A2A2	OR (95% CI)	*p*‐Value	GENO	A1A1	A1A2	A2A2	OR (95% CI)	*p*‐Value
1 month	2/30/137	1(50.0)	3(10.0)	3(2.2)	5.66 (1.61–19.93)	**6.9 × 10^−3^**	3/30/136	0(0.0)	3(10.0)	4(3.0)	2.17 (0.60–7.85)	**.239**
6 months	1/29/119	1(100.0)	9(31.0)	6(5.0)	8.79 (2.99–25.83)	**7.77 × 10^−5^**	2/26/121	1(50.0)	6(23.1)	9(7.4)	3.80 (1.43–10.11)	**7.55 × 10^−3^**
12 months	1/28/93	1(100.0)	10(35.7)	13(14.0)	4.10 (1.62–10.39)	**2.90 × 10^−3^**	2/23/97	2(100.0)	10(43.5)	12(12.4)	8.29 (2.87–23.89)	**9.08 × 10^−5^**
24 months	1/15/47	1(100.0)	10(66.7)	15(31.9)	4.34 (1.29–14.57)	**.017**	2/16/45	2(100.0)	11(68.8)	13(28.9)	9.31 (2.30–37.59)	**1.74 × 10^−3^**

*Note*: *UBASH3B* SNP rs2276408: A1 = T, A2 = C; *FBN2* SNP rs3805625: A1 = T, A2 = G.

Abbreviations: A1, minor allele; A2, non‐minor allele; CHR, chromosome; CI, confidence interval; *FBN2*, fibrillin 2; Func, the region of the genome where the mutation is; GENO, number of each genotype (A1A1/A1A2/A2A2); OR, odds ratio; SNP, single‐nucleotide polymorphism; *UBASH3B*, ubiquitin‐associated and SH3 domain containing B.

### 3.3 Effects of suggestive genetic variations on the pharmacodynamics of dabigatran

Considering the sample size and measurement points of our experiment, the genome‐wide *p*‐value significant threshold was adjusted to 1.0 × 10^−5^ for screening suggestive genetic variations on PD. As a result, 17 suggestive SNPs of 14 genes met the inclusion criteria. The related effects of genetic variations on PD are presented in Table 3 (Manhattan plots and Q–Q plots are shown in Figures S1 and S2). For anti‐FIIa activity, there were four suggestive SNPs, including *SNX7* rs9433747 (*p* = 1.70 × 10^−7^), *BRD4* rs11669901 (*p* = 2.90 × 10^−6^), *FLCN* rs3744124 (*p* = 4.77 × 10^−6^) associated with peak level and *UBAP1* rs1556439 (*p* = 6.69 × 10^−6^) associated with trough level. The minor allele carriers of all these SNPs had significantly higher PD levels. For APTT, *IGLV3‐12* rs2073451 (*p* = 8.50 × 10^−6^) was associated with peak level, and *LRRC8E* rs3745382 (*p* = 8.41 × 10^−6^) and *PTPLAD1* rs11539008 (*p* = 9.69 × 10^−6^) were associated with trough level. The minor allele of *IGLV3‐12* rs2073451 was significantly associated with a lower value, whereas the minor alleles of *LRRC8E* rs3745382 and *PTPLAD1* rs11539008 were both associated with the higher values. For PT, 10 SNPs were successfully screened, including one for peak level and nine for trough levels. Peak levels for the minor allele of *ZNF230* rs12753 were significantly higher (*p* = 1.97 × 10^−6^). The minor alleles of the remaining nine SNPs were significantly associated with higher trough values, containing *ANP32A* rs12904108 (*p* = 7.13 × 10^−7^), *SLC25A28* rs12252561 (*p* = 7.81 × 10^−7^), *ABCC2* rs2273697 and rs4148395 (both *p* = 3.10 × 10^−6^), *MYBPC1* rs11110942, rs3751246 and rs11110952 (all *p* = 4.46 × 10^−6^), *CEP170B* rs60001925 (*p* = 5.53 × 10^−6^) and *GYPA* rs145195209 (*p* = 9.24 × 10^−6^). Among the seven SNPs selected for anti‐FIIa activity and APTT, all had significant influences on more than one of PD indices (*p* < .05). For *ZNF230* rs12753 associated with peak PT, it was significantly associated with peak anti‐FIIa activity and APTT (*p* < 1 × 10^−3^). To evaluate the effect of these suggestive SNPs on bleeding events, GWA analyses were performed (Table S4), and only the minor allele carriers of *SNX7* rs9433747 had a significantly increased bleeding risk at the 24th month visit (OR = 4.76, 95% CI: 1.20–18.95, *p* = .027) with the same trends at visits on 1, 6 and 12 months.

**TABLE 3:** Effects of suggestive genetic variations on the pharmacodynamics of dabigatran

**Gene**	**SNP**	**Genotypes** **^a^**	**Peak PD levels**							**Trough PD levels**						
			**GENO** **^b^**	**FIIa (ng/ml)**	***p*‐Value**	**FAPTT (s)**	***p*‐Value**	**FPT (s)**	***p*‐Value**	**GENO**	**GIIa (ng/ml)**	***p*‐Value**	**GAPTT (s)**	***p*‐Value**	**GPT (s)**	***p*‐Value**
*SNX7*	rs9433747	A1A1	0/28/127	/	**1.70 × 10^−7^**	/	**1.88 × 10^−3^**	/	**6.41 × 10^−5^**	0/27/124	/	**0.025**	/	.582	/	.828
		A1A2		228.26 ± 161.58		54.12 ± 13.62		15.14 ± 2.48			90.39 ± 70.74		40.50 ± 8.62		12.80 ± 1.30	
		A2A2		133.62 ± 111.91		47.00 ± 13.37		13.86 ± 3.76			63.31 ± 56.42		39.35 ± 10.11		13.92 ± 6.67	
*BRD4*	rs11669901	A1A1	1/18/136	287.67	**2.90 × 10^−6^**	51.8	.096	13.60	.172	1/17/133	131.07	**0.015**	35.0	.665	11.10	.660
		A1A2		219.50 ± 172.25		52.03 ± 10.67		14.36 ± 2.25			98.71 ± 87.31		41.55 ± 8.01		12.83 ± 1.57	
		A2A2		140.56 ± 117.38		47.77 ± 14.01		14.06 ± 3.75			63.78 ± 54.43		39.33 ± 10.09		13.86 ± 6.45	
*FLCN*	rs3744124	A1A1	6/54/95	288.71 ± 193.56	**4.77 × 10^−6^**	65.37 ± 21.22	**6.05 × 10^−3^**	21.15 ± 12.97	.169	6/51/94	135.92 ± 104.60	**6.40E‐03**	52.30 ± 13.05	.064	17.73 ± 9.76	.729
		A1A2		150.23 ± 135.44		47.15 ± 13.86		13.81 ± 2.43			67.13 ± 60.37		37.45 ± 9.39		13.42 ± 5.71	
		A2A2		142.19 ± 111.94		47.86 ± 12.20		13.80 ± 1.97			64.39 ± 53.15		39.88 ± 9.22		13.63 ± 5.89	
*UBAP1*	rs1556439	A1A1	1/15/139	455.10	.117	66.30	.054	16.70	.063	1/14/136	263.48	**6.69E‐06**	45.80	**.011**	13.00	.083
		A1A2		182.90 ± 109.48		53.83 ± 14.08		14.79 ± 2.06			117.20 ± 88.94		46.56 ± 11.97		17.54 ± 11.03	
		A2A2		144.97 ± 126.69		47.56 ± 13.47		13.99 ± 3.73			61.67 ± 51.29		38.79 ± 9.35		13.33 ± 5.19	
*IGLV3‐12*	rs2073451	A1A1	30/74/51	120.01 ± 94.94	**.021**	43.33 ± 11.40	**8.50 × 10^−6^**	13.42 ± 1.91	**1.61 × 10^−4^**	29/75/47	63.12 ± 47.87	0.638	37.56 ± 8.39	.120	12.78 ± 1.82	.904
		A1A2		151.86 ± 134.17		47.54 ± 13.55		14.10 ± 4.64			68.02 ± 65.70		39.37 ± 10.16		13.54 ± 5.30	
		A2A2		166.76 ± 131.18		52.29 ± 13.98		14.47 ± 2.37			71.48 ± 57.36		41.06 ± 10.00		14.60 ± 8.42	
*LRRC8E*	rs3745382	A1A1	4/29/122	213.08 ± 107.13	**9.22 × 10^−3^**	50.10 ± 14.00	.067	13.70 ± 2.05	.282	3/28/120	184.95 ± 105.36	**1.74E‐03**	52.07 ± 19.68	**8.41 × 10^−6^**	16.90 ± 5.43	.143
		A1A2		172.76 ± 143.83		54.62 ± 15.02		15.33 ± 6.85			78.56 ± 69.50		45.13 ± 10.62		15.17 ± 8.64	
		A2A2		143.35 ± 122.89		46.72 ± 12.88		13.80 ± 2.17			62.81 ± 52.45		37.94 ± 8.53		13.30 ± 5.25	
*PTPLAD1*	rs11539008	A1A1	0/22/133	/	.209	/	**3.09 × 10^−3^**	/	**.012**	0/21/130	/	**1.41E‐03**	/	**9.69 × 10^−6^**	/	**.024**
		A1A2		162.47 ± 135.75		54.38 ± 16.79		14.80 ± 2.81			105.75 ± 92.08		47.90 ± 38.20		17.63 ± 11.98	
		A2A2		148.56 ± 126.02		47.28 ± 12.83		13.97 ± 3.70			62.08 ± 50.65		10.65 ± 9.04		13.09 ± 4.12	
*ZNF230*	rs12753	A1A1	11/52/92	144.02 ± 176.03	**1.86 × 10^−4^**	48.35 ± 19.92	**2.99 × 10^−4^**	14.84 ± 3.28	**1.97 × 10^−6^**	10/49/92	50.26 ± 65.69	0.414	37.10 ± 9.68	.820	12.75 ± 0.97	.760
		A1A2		156.90 ± 126.10		50.95 ± 13.29		14.23 ± 2.12			82.24 ± 73.63		40.16 ± 9.34		14.38 ± 8.21	
		A2A2		147.77 ± 121.26		46.78 ± 12.73		13.92 ± 4.23			62.60 ± 49.07		39.50 ± 10.12		13.48 ± 4.95	
*ANP32A*	rs12904108	A1A1	5/41/109	103.61 ± 108.62	.841	47.04 ± 12.89	.391	13.20 ± 1.58	.277	5/40/106	56.31 ± 27.70	0.477	42.04 ± 9.95	.143	26.82 ± 18.10	**7.13 × 10^−7^**
		A1A2		162.36 ± 137.36		49.73 ± 10.94		14.25 ± 2.23			77.40 ± 65.59		41.22 ± 8.57		14.15 ± 6.91	
		A2A2		148.36 ± 123.95		47.80 ± 14.59		14.07 ± 4.05			65.23 ± 58.69		38.81 ± 10.23		12.94 ± 3.25	
*SLC25A28*	rs12252561	A1A1	9/44/102	173.09 ± 85.29	.796	54.33 ± 10.10	.291	14.34 ± 1.47	.219	8/43/100	105.80 ± 71.81	0.226	47.69 ± 10.39	**.0499**	23.50 ± 15.12	**7.81 × 10^−7^**
		A1A2		129.74 ± 101.74		48.70 ± 10.35		13.91 ± 1.75			69.65 ± 73.56		40.56 ± 8.22		13.92 ± 6.59	
		A2A2		157.56 ± 139.00		47.57 ± 15.03		14.14 ± 4.26			64.50 ± 50.93		38.47 ± 10.13		12.85 ± 3.26	
*ABCC2*	rs2273697	A1A1	3/21/131	209.74 ± 74.86	.598	51.83 ± 11.05	.441	14.20 ± 1.36	.426	2/21/128	98.32 ± 10.57	0.052	44.70 ± 6.60	.286	32.10 ± 19.20	**3.10 × 10^−6^**
		A1A2		161.40 ± 104.82		49.48 ± 9.88		13.84 ± 1.87			95.65 ± 96.79		42.03 ± 11.47		17.13 ± 11.46	
		A2A2		147.55 ± 131.37		48.01 ± 14.24		14.13 ± 3.84			63.17± 50.64		39.07 ± 9.54		12.88 ± 2.92	
*ABCC2*	rs4148395	A1A1	3/21/131	209.74 ± 74.86	.598	51.83 ± 11.05	.441	14.20 ± 1.36	.426	2/21/128	98.32 ± 10.57	0.052	44.70 ± 6.60	.286	32.10 ± 19.20	**3.10 × 10^−6^**
		A1A2		161.40 ± 104.82		49.48 ± 9.88		13.84 ± 1.87			95.65 ± 96.79		42.03 ± 11.47		17.13 ± 11.46	
		A2A2		147.55 ± 131.37		48.01 ± 14.24		14.13 ± 3.84			63.17 ± 50.64		39.07 ± 9.54		12.88 ± 2.92	
*MYBPC1*	rs11110942	A1A1	7/61/87	112.94 ± 83.09	.739	50.53 ± 6.80	.708	13.77 ± 1.03	.776	7/59/85	67.07 ± 49.26	0.239	44.83 ± 9.39	.292	24.00 ± 17.42	**4.46 × 10^−6^**
		A1A2		146.66 ± 107.43		47.03 ± 10.69		13.75 ± 1.90			75.23 ± 72.71		39.38 ± 9.01		13.85 ± 5.41	
		A2A2		156.34 ± 141.97		48.99 ± 15.75		14.35 ± 4.51			63.34 ± 49.96		39.24 ± 10.35		12.79 ± 3.31	
*MYBPC1*	rs3751246	A1A1	7/62/86	112.94 ± 83.09	.739	50.53 ± 6.80	.708	13.77 ± 1.03	.776	7/60/84	67.07 ± 49.26	0.270	44.83 ± 9.39	.240	24.00 ± 17.42	**4.46 × 10^−6^**
		A1A2		147.79 ± 106.92		47.22 ± 10.71		13.79 ± 1.90			75.13 ± 72.11		39.59 ± 9.08		13.87 ± 5.37	
		A2A2		155.65 ± 142.63		48.87 ± 15.80		14.33 ± 4.53			63.26 ± 50.26		39.08 ± 10.31		12.76 ± 3.32	
*MYBPC1*	rs11110952	A1A1	7/63/85	112.94 ± 83.09	.739	50.53 ± 6.80	.708	13.77 ± 1.03	.776	7/60/84	67.07 ± 49.26	0.270	44.83 ± 9.39	.240	24.00 ± 17.42	**4.46 × 10^−6^**
		A1A2		147.07 ± 106.22		47.35 ± 10.68		13.81 ± 1.89			75.13 ± 72.11		39.59 ± 9.08		13.87 ± 5.37	
		A2A2		156.26 ± 143.35		48.79 ± 15.88		14.32 ± 4.56			63.26 ± 50.26		39.08 ± 10.31		12.76 ± 3.32	
*CEP170B*	rs60001925	A1A1	3/34/118	109.85 ± 14.90	.852	58.70 ± 5.77	.443	14.43 ± 1.81	.400	3/30/118	32.15 ± 21.05	0.481	44.93 ± 2.95	.113	24.67 ± 15.45	**5.53 × 10^−6^**
		A1A2		147.58 ± 132.20		49.34 ± 12.49		14.24 ± 2.09			71.14 ± 55.42		41.79 ± 10.38		15.79 ± 10.33	
		A2A2		152.50 ± 127.65		47.72 ± 14.03		14.04 ± 3.96			68.31 ± 61.65		38.85 ± 9.72		12.92 ± 3.08	
*GYPA*	rs145195209	A1A1	2/21/132	110.87 ± 8.19	.280	71.35 ± 17.55	.604	15.70 ± 3.30	.818	1/22/128	60.08	0.601	46.50	.920	46.50	**9.24 × 10^−6^**
		A1A2		148.31 ± 163.25		44.61 ± 11.77		13.60 ± 1.94			63.45 ± 75.96		39.28 ± 9.41		14.66 ± 8.22	
		A2A2		151.59 ± 121.54		48.52 ± 13.53		14.14 ± 3.79			69.03 ± 57.18		39.54 ± 9.97		13.30 ± 4.84	

Abbreviations: A1, minor allele; A2, non‐minor allele; GENO, number of each genotype (A1A1/A1A2/A2A2); SNP, single‐nucleotide polymorphism.

^a^ *SNX7* SNP rs9433747: A1 = G, A2 = A; *BRD4* SNP rs11669901: A1 = A, A2 = G; *FLCN* SNP rs3744124: A1 = T, A2 = C; *UBAP1* SNP rs1556439: A1 = T, A2 = C; *IGLV3‐12* SNP rs2073451: A1 = G, A2 = A; *LRRC8E* SNP rs3745382: A1 = A, A2 = G; *PTPLAD1* SNP rs11539008: A1 = A, A2 = G; *ZNF230* SNP rs12753: A1 = A, A2 = C; *ANP32A* SNP rs12904108: A1 = T, A2 = A; *SLC25A28* SNP rs12252561: A1 = C, A2 = G; *ABCC2* SNP rs2273697: A1 = A, A2 = G; *ABCC2* SNP rs4148395: A1 = A, A2 = G; *MYBPC1* SNP rs11110942: A1 = G, A2 = C; *MYBPC1* SNP rs3751246: A1 = T, A2 = C; *MYBPC1* SNP rs11110952: A1 = C, A2 = T; *CEP170B* SNP rs60001925: A1 = C, A2 = CGCAGGA; *GYPA* SNP rs145195209: A1 = A, A2 = AT.

^b^ As the number of peak anti‐FIIa activity was 156 and the number of peak APTT and PT was 155, the genotype of extra one that had only peak anti‐FIIa activity was: *SNX7* SNP rs9433747: A2A2; *BRD4* SNP rs11669901: A2A2; *FLCN* SNP rs3744124: A2A2; *UBAP1* SNP rs1556439: A2A2; *IGLV3‐12* SNP rs2073451: A1A2; *LRRC8E* SNP rs3745382: A2A2; *PTPLAD1* SNP rs11539008: A1A2; *ZNF230* SNP rs12753: A1A2; *ANP32A* SNP rs12904108: A2A2; *SLC25A28* SNP rs12252561: A2A2; *ABCC2* SNP rs2273697: A2A2; *ABCC2* SNP rs4148395: A2A2; *MYBPC1* SNP rs11110942: A2A2; *MYBPC1* SNP rs3751246: A2A2; *MYBPC1* SNP rs11110952: A2A2; *CEP170B* SNP rs60001925: A2A2; *GYPA* SNP rs145195209: A1A2.

### 3.4 Candidate gene association analysis

Except for the three candidate genes not detected (*CES1P2*, *CYP3A4* and *UGT2B15*), correlation analyses were performed for the remaining 22 reported genes and 148 detected SNPs, and we identified a total of 14 candidate genes and 33 new SNPs. The impacts of reported genes on bleeding and PD of dabigatran are summarized in Table 4 and Table S5. A total of six genes and 10 SNPs showed an effect on bleeding, including *ABCG2* rs2231165, *CYP2A6* rs8192720 and rs8192726, *CYP2B6* rs3745276 and rs3745277, *CYP2J2* rs3738474, *FRAS1* rs17003071, *SLCO1B1* rs2291076, rs2306283 and rs4149032 (*p* < .05). Among these SNPs, *ABCG2* rs2231165, *CYP2A6* rs8192720 and *SLCO1B1* rs4149032 were associated with bleeding at more than one visit. A total of 11 genes and 25 SNPs had the positive effects on PD (*p* < .05), except three genes (*CYP2A6*, *CYP2J2* and *SLCO1B1*). There were two genes and six SNPs (*ABCG2* rs2231142, rs2231148 and rs2231156, *CES1* rs112236246, rs2244614 and rs3217164) associated with more than one PD parameter. Only one SNP (*ABCG2* rs2231156) was identified for the association with both bleeding and PD. For reported *CES1* rs2244613 associated with bleeding, no association was found in our study. Further, these reported SNPs associated with PD were also found to be negative in our study, including *ABCG2* rs2231138, *CYP2B6* rs2279342, *CYP2C19* rs12769205, rs3758580 and rs4244285, *CYP3A5* rs15524 and rs4646453, *FRAS1* rs6835769, *SLC4A4* rs138389345, *SLCO1B1* rs11045748, *SULT1A1* rs9282862 and *UGT1A9* rs12466997.

**TABLE 4:** Positive effects of candidate genes on bleeding and the pharmacodynamics of dabigatran

**SNP**	**Gene**	**Bleeding 1 month**	**Bleeding 6 months**	**Bleeding 12 months**	**Bleeding 24 months**	**FIIa (ng/ml)**	**FAPTT (s)**	**FPT (s)**	**GIIa (ng/ml)**	**GAPTT (s)**	**GPT (s)**
		***p*‐Values**									
rs2235013^a^	*ABCB1*	0.102	0.572	0.396	0.112	**0.021**	0.087	0.094	0.496	0.430	0.786
rs2235033^a^	*ABCB1*	0.102	0.572	0.396	0.112	**0.021**	0.087	0.094	0.496	0.430	0.786
rs2273697^a^	*ABCC2*	/	0.249	0.701	0.990	0.598	0.441	0.426	0.052	0.286	**3.10E‐06**
rs4148395^a^	*ABCC2*	/	0.249	0.701	0.990	0.598	0.441	0.426	0.052	0.286	**3.10E‐06**
rs2231142^b^	*ABCG2*	0.352	0.691	0.273	0.227	0.111	**0.017**	**0.016**	**7.21E‐03**	**1.79E‐03**	0.705
rs2231148^a^	*ABCG2*	0.683	0.568	0.892	0.960	0.201	0.083	**0.032**	**0.028**	0.811	0.129
rs2231156^a^	*ABCG2*	0.165	0.823	0.782	0.457	0.222	**0.030**	0.118	**5.96E‐04**	**3.91E‐03**	0.559
rs2231165^a^	*ABCG2*	0.242	**7.07E‐03**	0.083	**0.049**	0.921	0.219	0.823	0.605	**0.021**	0.457
rs112236246^a^	*CES1*	0.758	0.902	0.427	0.454	0.322	0.283	0.783	0.150	**0.028**	**0.027**
rs2244614^a^	*CES1*	0.763	0.910	0.441	0.348	0.342	0.459	0.925	0.157	**0.040**	**0.025**
rs3217164^a^	*CES1*	0.763	0.910	0.441	0.348	0.342	0.459	0.925	0.157	**0.040**	**0.025**
rs4646427^a^	*CYP1A2*	0.259	0.084	0.435	0.449	0.161	0.097	**0.037**	0.517	0.625	0.558
rs8192720^a^	*CYP2A6*	0.222	0.081	**0.017**	**0.030**	0.621	0.905	0.788	0.697	0.958	0.340
rs8192726^a^	*CYP2A6*	0.441	0.797	0.136	**0.018**	0.293	0.584	0.944	0.457	0.142	0.605
rs8192719^a^	*CYP2B6*	0.667	0.991	0.632	0.944	0.286	0.931	0.708	**0.023**	0.419	0.654
rs3745275^a^	*CYP2B6, CYP2A13*	0.690	0.419	0.183	0.210	**0.014**	0.602	0.274	0.251	0.156	0.904
rs3745276^a^	*CYP2B6, CYP2A13*	0.494	0.172	**0.028**	0.068	0.643	0.850	0.840	0.795	0.306	0.647
rs3745277^a^	*CYP2B6, CYP2A13*	**0.041**	0.141	0.159	0.984	0.459	**0.048**	0.119	0.696	0.738	0.832
rs7249735^a^	*CYP2B6, CYP2A13*	0.835	0.378	0.246	0.122	**0.011**	0.638	0.240	0.124	0.270	0.945
rs1058932^a^	*CYP2C8*	0.580	0.235	0.126	0.099	0.515	0.832	0.603	0.148	0.923	**0.011**
rs11572078^a^	*CYP2C8*	0.580	0.235	0.126	0.099	0.515	0.832	0.603	0.148	0.923	**0.011**
rs2275622^a^	*CYP2C8*	0.983	0.628	0.241	0.208	0.339	0.882	0.875	0.259	0.997	**0.016**
rs17847029	*CYP2C9*	0.141	0.813	0.701	0.453	**0.043**	0.083	0.088	0.931	0.882	0.860
rs3738474	*CYP2J2*	**0.035**	0.111	0.234	0.538	0.940	0.809	0.718	0.854	0.648	0.737
rs11937525	*FRAS1*	0.184	0.334	0.819	0.855	0.289	0.259	0.120	0.182	0.398	**0.023**
rs17003071	*FRAS1*	0.945	0.704	**0.0492**	0.199	0.566	0.893	0.897	0.713	0.465	0.298
rs17003160	*FRAS1*	0.261	0.753	0.759	0.680	0.633	0.553	0.538	0.678	0.935	**0.028**
rs398092530	*FRAS1*	0.242	0.858	0.619	0.552	0.569	0.532	0.381	0.923	0.919	**0.039**
rs1062677	*SLC4A4*	/	0.713	0.456	0.735	0.441	0.715	0.764	**0.022**	0.200	0.765
rs2291076^a^	*SLCO1B1*	0.121	0.166	0.193	**0.043**	0.402	0.523	0.897	0.112	0.589	0.617
rs2306283^a^	*SLCO1B1*	0.121	0.154	0.164	**0.043**	0.686	0.826	0.854	0.129	0.634	0.685
rs4149032^a^	*SLCO1B1*	0.471	0.217	**0.047**	**0.022**	0.924	0.660	0.899	0.138	0.274	0.085
rs79527462	*SULT1A1*	0.599	0.999	0.527	0.176	0.083	0.471	0.053	**0.013**	0.179	0.630

Abbreviations: *ABCB1*, ATP‐binding cassette subfamily B member 1; *ABCC2*, ATP‐binding cassette subfamily C member 2; *ABCG2*, ATP binding cassette subfamily G member 2; *CES1*, carboxylesterase 1; *CYP1A2*, cytochrome P450 family 1 subfamily A member 2; *CYP2A6*, cytochrome P450 family 2 subfamily A member 6; *CYP2B6*, cytochrome P450 family 2 subfamily B member 6; *CYP2A13*, cytochrome P450 family 2 subfamily A member 13; *CYP2AJ2*, cytochrome P450 family 2 subfamily J member 2; *CYP2C8*, cytochrome P450 family 2 subfamily C member 8; *CYP2C9*, cytochrome P450 family 2 subfamily C member 9; *SLC4A4*, solute carrier family 4 member 4; *SLCO1B1*, solute carrier organic anion transporter family member 1B1; *SULT1A1*, sulfotransferase family 1A member 1.

^a^ The SNP was detected and analyzed for variations associated with the pharmacodynamics in reported pharmacogenomic studies of dabigatran.

^b^ The SNP was detected and analyzed for variations associated with bleeding in reported pharmacogenomic studies of dabigatran.

## 4 DISCUSSION

We performed a nationwide multicentre prospective cohort study and genome‐wide pharmacogenetic analysis to identify the genetic variations on bleeding and PD of dabigatran in Chinese patients with NVAF. As far as we are aware, this is the first GWA study focusing on Chinese NVAF populations with dabigatran. Using whole‐exome sequencing and correlation analysis, we first screened two suggestive SNPs (*UBASH3B* rs2276408 and *FBN2* rs3805625) associated with bleeding, which revealed that the minor allele carriers had higher bleeding risks at the sixth and 12th month visits, respectively. Moreover, the same significant trends for these SNPs were also presented at other visits. Second, there were four (*SNX7* rs9433747, *BRD4* rs11669901, *FLCN* rs3744124 and *UBAP1* rs1556439), three (*IGLV3‐12* rs2073451, *LRRC8E* rs3745382 and *PTPLAD1* rs11539008) and 10 (*ZNF230* rs12753, *ANP32A* rs12904108, *SLC25A28* rs12252561, *ABCC2* rs2273697 and rs4148395, *MYBPC1* rs11110942, rs3751246 and rs11110952, *CEP170B* rs60001925 and *GYPA* rs145195209) SNPs that successfully exceeded the threshold to affect anti‐FIIa activity, APTT, and PT, respectively. Apart from the fact that the minor allele carriers of *IGLV3‐12* rs2073451 show a lower PD value, the minor allele carriers of remaining 16 SNPs had higher PD levels. Lastly, we discovered 33 new relevant SNPs of 14 reported genes linked to bleeding and PD. A total of six genes (*ABCG2*, *CYP2A6*, *CYP2B6*, *CYP2J2*, *FRAS1* and *SLCO1B1*) affected bleeding and 11 genes (*ABCB1*, *ABCC2*, *ABCG2*, *CES1*, *CYP1A2*, *CYP2B6*, *CYP2C8*, *CYP2C9*, *FRAS1*, *SLC4A4* and *SULT1A1*) influenced PD. For the reported positive SNPs associated with bleeding or PD parameters, we found the opposite results in our study. As we collected as many indicators as possible and prolonged follow‐up time, our results deserved to be understandable. Our integrated studies, including previous similar study on healthy participants,^22^ have comprehensively explored genetic variations associated with PK, PD and clinical outcomes in the Chinese populations treated with dabigatran.

Considering high inter‐ and intraindividual CVs of dabigatran as well as the greater risk of stroke/SE events, major bleeding and GI bleeding,^29^ many studies have focused on exploring biomarkers on treatment. The prior trial showed that dabigatran's higher trough concentrations were linked to increased bleeding and decreased thromboembolic events.^10^ The predicted peak and trough plasma levels of dabigatran in individuals with AF were 52–383 and 28–215 ng/ml, respectively, according to a European guideline.^30^ However, PK measurement methods like high‐performance liquid chromatography or mass spectrometry took a long time, and the new method of chromogenic assay was not available in all areas. Therefore, PD indices determined qualitatively and quantitatively might be more conveniently accessible. Traditional coagulation indicators, including PT and APTT, were generally prolonged in a concentration‐dependent pattern for dabigatran patients.^30^, ^31^, ^32^, ^33^ Anti‐FIIa activity, as a specific test determined by chromogenic assay, has been recommended, and it demonstrated that peak anti‐FIIa activity could be an indicator for predicting bleeding outcomes of dabigatran.^32^, ^33^ Consequently, our study focused on genetic variation on the direct outcomes of bleeding and indirect outcomes of PD, including anti‐FIIa activity, APTT and PT.

The *UBASH3B* gene (ubiquitin‐associated and SH3 domain containing B, also known as TULA‐2, TULA2) is a protein‐coding gene and located in chromosome 11q24.1. The rs2276408 is intron variant of *UBASH3B*, which has a C base that becomes a T base (C>T). From the 1000 Genomes Project Phase 3 populations data, MAF (T) is 21% in all the populations, including 32% in American and 10% in East Asian. From the HPA RNA‐seq normal tissue data, *UBASH3B* gene mainly expresses in human spleen and placenta, as well as a small amount of distribution in kidney and liver.^34^ These proteins encoded by this gene have the functions of preventing platelet‐derived growth factor receptor and epidermal growth factor receptor from being endocytosed. Previous studies have demonstrated that heparin‐induced thrombocytopenia and adverse drug reaction pathways were associated with *UBASH3B* gene.^35^, ^36^ The sole member of the T‐cell ubiquitin ligand (TULA) family among the protein tyrosine phosphatases found in the platelets of both mice and humans is TULA‐2.^37^ This gene encodes this protein. As the platelet Fcγ‐receptor (FcγRIIa) is a causative factor of thrombin generation and thrombotic complications,^38^ TULA‐2 may interfere with the platelet FcγRIIa cascade by dephosphorylating Syk. An in vivo study in mice revealed that lower levels of TULA‐2 enhanced platelet reactivity, worsened thrombocytopenia and thrombosis and reduced the time it took for tails to bleed.^35^ Although dabigatran inhibits thrombin factor in coagulation cascade, thrombin also participates in platelet aggregation and finally results in thrombosis formation.^39^ In our study, MAF (T) of *UBASH3B* rs2276408 is 10.0%, which is consistent with reported data. At the the sixth month visit, minor allele carriers had a significantly greater incidence rate of bleeding than noncarriers (TT 100.0%, TC 31.0% and CC 5.0%). Moreover, the trends in other visits of 1, 12 and 24 months also showed similar results. Besides, the minor allele carriers had a higher peak anti‐FIIa activity than noncarriers (TT 330.93 ± 44.08 ng/ml, TC 194.21 ± 145.66 ng/ml and CC 137.61 ± 119.31 ng/ml).

The *FBN2* gene (fibrillin 2) is also a protein‐coding gene and located in chromosome 5q23.3. A G base is converted into a T base (G>T) in the rs3805625 mutation, which causes intron variation. From the 1000 Genomes Project Phase 3 populations data, MAF (T) is 2% in all the populations. Interestingly, it is almost expressed only in East Asian population (9%). In other populations, the T frequency is almost 0%, especially no mutations in American and African populations. From the HPA RNA‐seq normal tissue data, the expression level of *FBN2* gene in human placenta is much higher, with very little in kidney and liver.^40^ Whether mutations in this gene cause congenital contractural arachnodactyly remains controversy.^41^, ^42^ The extracellular matrix structural ingredient and calcium ion binding are gene ontology annotations connected to this gene. *FBN1* (fibrillin 1), a significant paralog of this gene, has mutations linked to Marfan syndrome. The extracellular matrix gp (fibrillin‐1) encoded by *FBN1* gene is a structural element of calcium‐binding microfilaments. It might mediate cell adhesion by interacting with the cell surface receptors integrins ITGAV:ITGB3 and ITGA5:ITGB1,^43^, ^44^ and subsequently bind heparin, resulting in assembly of microfibrils.^45^ *FBN1* gene involvement in the pathways of platelet activation was discovered in a recent work focused on a multilayer systems biology investigation of gastric cancer.^46^ Besides, activated fibrinolysis, thrombin and platelet dysfunction are less well known but are indeed important features of Marfan syndrome.^47^ The above information may provide us preliminary insight into exploring the effect of *FBN2* gene on bleeding of drugs. According to our results, the MAF (T) of *FBN2* rs3805625 is 10.6%, which is consistent with reported data. At the 12th month visit, the bleeding rates in minor allele carriers were significantly higher than noncarriers (TT 100.0%, TG 43.5% and GG 12.4%). Moreover, these increased trends were also presented in other visits on the 6th and 24th month.

For suggestive genes and SNPs associated with PD, the detailed characteristics are shown in Table S6. Most genes express in kidney and liver except *IGLV3‐12* and *MYBPC1*. The MAFs of these SNPs in our study are almost consistent with that in East Asian population. Interestingly, MAFs of some SNPs in East Asian population were higher or lower compared with those in American and European populations. To our knowledge, all these genes are protein‐coding genes, and some genes have demonstrated the therapeutic potential in cardiovascular disease.^48^, ^49^ There is, however, little knowledge of how these genes and SNPs affect platelet or coagulation function. Previous pharmacoproteomic investigation in a standardized murine model found that the inductive effect of exosomes, which were abundant with coagulation factors, might be decreased by BRD4 inhibitors.^50^ Besides, a rat study revealed that a BRD4 inhibitor might reduce clinical platelet counts.^51^ Glycophorins A (GYPA) and B are the major sialoglycoproteins of human erythrocyte membrane. Response to elevated platelet cytosolic Ca^2+^ is the related pathway of *GYPA* gene. According to the study on coronary high‐signal intensity plaques (HIPs), which was immunoreactive for GYPA and fibrin, intraplaque bleeding occurred much more in HIPs than in non‐HIPs.^52^ The *ABCC2* gene mainly encodes multidrug‐resistance‐associated protein 2, and previous studies of genetic variation on dabigatran showed different results.^18^, ^22^ Our similar study in 118 Chinese healthy participants revealed *ABCC2* rs2273697 and rs4148395 affected AUC, and *C*_max_ as well as rs717620 had no influence on PK and PD.^22^ Another study in 107 Spanish healthy participants found there was no association between *ABCC2* rs2273697 and rs717620 and PK.^18^ In this study, which focused on Chinese patients with NVAF, the minor allele (A) carriers of *ABCC2* rs2273697 and rs4148395 had a higher trough PT than noncarriers (AA 32.10 ± 19.20 s, AG 17.13 ± 11.46 s and GG 12.88 ± 2.92 s).

Previous pharmacogenomic studies about the association between *CES1* rs2244613 and bleeding of dabigatran in patients with AF was controversial. Two studies performed in European and Chinese patients^20^, ^21^ showed that the allele (C) carriers had a lower risk of bleeding, especially minor bleeding. On the other hand, other two studies^19^, ^53^ as well as our results found no association. Besides, another SNP, *ABCB1* rs1045642, which was reported the allele (T) carriers had an increased risk of bleeding in patients after total knee arthroplasty with dabigatran,^54^ had no association with bleeding in patients with AF, including our results. Further, our result of *ABCG2* rs2231142 was negative for bleeding, which was consistent with previous study.^19^ Our identified six genes with bleeding were only explored for association with PK/PD in previous studies of healthy participants, and most had negative results except *SLCO1B1* rs4149032 associated with maximum plasma concentration.^22^ There were no associations detected in previous studies for our identified 11 genes and 25 SNPs associated with PD. Besides, all these reported positive SNPs with PD had negative outcomes in our study.^22^ We found *ABCG2* rs2231156 had a significant impact on bleeding and PD; however, the prior study only discovered the positive result in time to peak concentration of healthy participants.^22^

Some limitations in our study must be discussed. First, although the sample size of our study was larger than few other researches (Table S1), the number of patients included (*n* = 170) was still limited, and our results should be verified in other studies with more patients. Second, the dabigatran regimen was 110 mg twice daily for all and might decrease bleeding events. The reasons were considered that the formulations of 150 and 75 mg were approved later than that of 110 mg in China and the reduced‐dose anticoagulants were more used in Asian population.^55^ Consequently, more different doses should be included in future studies. Third, the incidence rates of TEs and MACEs were relatively low. This might be also associated with sample size as well as loss to follow‐up. Future studies should be performed to ensure completion of visits. Fourth, considering the protection of participants from the perspective of the ethics committee, PK parameters were not included. Peak and trough plasma levels of dabigatran might be also meaningful for predicting bleeding risk, and the technology of concentration measurement should be improved in future. Finally, the mechanisms of thrombosis and haemorrhage in our new‐found suggestive genes and SNPs were not well understood. More future in vivo and in vitro pharmacogenomic and proteomic studies that explore and validate our results are required. Together with shortcomings that we already mentioned, we suggested that overall results of this explorative study should be more circumspective.

## 5 CONCLUSIONS

Genetic variations have a potential influence on bleeding risk and PD of dabigatran in Chinese patients with NVAF. The suggestive two SNPs (*UBASH3B* rs2276408 and *FBN2* rs3805625) associated with bleeding as well as 17 new SNPs of 14 genes (*SNX7*, *BRD4*, *FLCN*, *UBAP1*, *IGLV3‐12*, *LRRC8E*, *PTPLAD1*, *ZNF230*, *ANP32A*, *SLC25A28*, *ABCC2*, *MYBPC1*, *CEP170B* and *GYPA*) associated with PD may be novel targets for anticoagulation therapy. Considering the explorative nature of our study, how the functions of these SNPs on mechanism of anticoagulant work, as well as whether these genetic variations affect other populations or outcomes of dabigatran, must be investigated in future via in vitro and in vivo researches.

## CONFLICT OF INTEREST

The authors declare that the research was conducted in the absence of any commercial or financial relationships that could be construed as a potential conflict of interest.

## Supporting information

Table S1 Previous pharmacogenomic studies and candidate genes reported for dabigatran
Table S2 Detailed clinical outcomes of patients treated with dabigatran at all follow‐up visits
Table S3 Effects of *UBASH3B* rs2276408 and *FBN2* rs3805625 on the pharmacodynamics of dabigatran
Table S4 Effects of suggestive SNPs associated with PD parameters on bleeding events of patients treated with dabigatran
Table S5 Negative effects of candidate genes on bleeding and the pharmacodynamics of dabigatran
Table S6 Characteristics of suggestive genes and SNPs associated with PD parameters

Figure S1 Manhattan plots of association with pharmacodynamic parameters
Figure S2 Quantile–quantile plots of association with pharmacodynamic parameters

## Data Availability

The data presented in the study are deposited in the National Population Health Data Center (NPHDC) repository, accession number 10.12213/11.A0028.202009.338.V1.0 (https://www.ncmi.cn/phda/dataDetails.html?type=project_data&id=CSTR:A0006.11.A0028.202009.338.V1.0‐V1.0).
