# Supplementary material for: Genetic variations in relation to bleeding and pharmacodynamics of dabigatran in Chinese patients with nonvalvular atrial fibrillation: A nationwide multicentre prospective cohort study
Source: Clin Transl Med. 2022 Dec 1;12(12):e1104. doi: 10.1002/ctm2.1104 (PMC9714378; doi:10.1002/ctm2.1104)
Supplement: Supplementary file 1 — Table S1 Previous pharmacogenomic studies and candidate genes reported for dabigatran Table S2 Detailed clinical outcomes of patients treated with dabigatran at all follow‐up visits Table S3 Effects of UBASH3B rs2276408 and FBN2 rs3805625 on the pharmacodynamics of dabigatran Table S4 Effects of suggestive SNPs associated with PD parameters on bleeding events of patients treated with dabigatran Table S5 Negative effects of candidate genes on bleeding and the pharmacodynamics of dabigatran Table S6 Characteristics of suggestive genes and SNPs associated with PD parameters [file CTM2-12-e1104-s002.docx]

**Supplementary 1. Tables**

**Table S1.** **Previous pharmacogenomic studies and candidate genes reported of dabigatran**

| **Studies** | **Sample size** | **Age** | **Male**  **n(%)** | **Weight** | **Ethnicity** | **Genes** | **SNPs** | **PK/PD and Clinical Outcomes** | |
| --- | --- | --- | --- | --- | --- | --- | --- | --- | --- |
|  |  |  |  |  |  |  |  | **Indicators** | **Significant effects** |
| **I. Healthy volunteers** | | | | | | | | | |
| Gouin-Thibault I et al, 2017 [1] | 60 | 28.0±9.2^a^ | 60(100.0) | 75.2±11.0^a^ | Caucasian | *ABCB1* | rs1045642, rs2032582, rs1128503 | AUC_0-∞_, C_max_, T_max_, CV% | No significant effect. |
|  |  |  |  |  |  | *CES1* | rs2244613 |  |  |
| Zubiaur P et al, 2020 [2] | 107 | 30.4±8.6 | 52(48.6) | 70.2±11.3 | Caucasian, Latin-American | *ABCB1* | rs1045642, rs2032582, rs1128503, rs3842, rs10276036, rs7787082, rs4728709, rs10248420, rs10280101, rs12720067, rs11983225, rs4148737 | AUC/DW, C_max_ /DW, T_max_, t_½_,, Vd/F, Cl/F | 1.*CYP2D6* poor metabolizers were related to lower Cl/F (p = 0.049) and a tendency was observed towards higher AUC (p = 0.07), C_max_ (p = 0.062) and to lower Vd/F (p = 0.08).  2.*SLC22A1* haplotype was related to pharmacokinetic variability (p < 0.05).  3. *CYP3A5*-expressing subjects (*1/*1) were related to a higher t_1/2_ compared to *1/*3 and *3/*3 (ANOVA, p = 0.04, after Bonferroni post hoc, p = 0.09) |
|  |  |  |  |  |  | *ABCC2* | rs2273697, rs717620 |  |  |
|  |  |  |  |  |  | *CES1* | rs2244613, rs71647871, rs8192935 |  |  |
|  |  |  |  |  |  | *CYP1A2* | rs2069514, rs762551, rs2470890 |  |  |
|  |  |  |  |  |  | *CYP2A6* | rs28399433 |  |  |
|  |  |  |  |  |  | *CYP4F2* | rs2108622 |  |  |
|  |  |  |  |  |  | *CYP2B6* | rs3745274, rs3211371, rs32113719, rs2279345, rs2279343 |  |  |
|  |  |  |  |  |  | *CYP2C8* | rs11572103, rs10509681, rs1058930 |  |  |
|  |  |  |  |  |  | *CYP2C9* | rs1799853, rs1057910 |  |  |
|  |  |  |  |  |  | *CYP2C19* | rs4244285, rs4986893, rs28399504, rs12248560 |  |  |
|  |  |  |  |  |  | *CYP2D6* | rs35742686, rs3892097, rs5030655, rs5030867, rs5030865, rs5030656, rs1065852, rs5030865, rs28371706, rs28371725 |  |  |
|  |  |  |  |  |  | *CYP3A4* | rs35599367, rs55785340, rs4646438 |  |  |
|  |  |  |  |  |  | *CYP3A5* | rs776746, rs10264272 |  |  |
|  |  |  |  |  |  | *SLCO1B1* | rs4149056, rs2306283, rs4149015, rs11045879 |  |  |
|  |  |  |  |  |  | *SLC22A1* | rs72552763, rs12208357, rs34059508 |  |  |
|  |  |  |  |  |  | *UGT1A1* | rs887829 |  |  |
| Liu Y et al, 2021 [3] | 106 | 31.6±6.9^a^ | 80(75.5) | 66.3±8.6^a^ | Chinese | *ABCB1* | rs1045642, rs4148738, rs2032582 | C_max_, T_max_, t_½_, AUC_last_, AUC_0-∞_, DW ratio, AUC/DW, C_max_ /DW | Under fasting condition, there was no significant effect. Under fed condition, *CES1* SNP rs8192935 was associated with C_max_ and t_½_. *ABCB1* rs1045642 had a significant effect on T_max_. P values were all not shown in paper. |
|  |  |  |  |  |  | *CES1* | rs2244613, rs8192935 |  |  |
| Xie QF et al, 2022 [4] | 118 | 24.0±5.6^a^ | 85(72.0) | NA | Chinese | *SLC4A4* | rs138389345 | AUC_0–t_, C_max_, T_max_, t_1_*_/_*_2_, anti-IIa activity2h, APTT2h, PT2h | *SLC4A4* SNP rs138389345 (p = 5.99 × 10**^–^**^5^), *FRAS1* SNP rs6835769 (p = 6.88 × 10**^–^**^5^), and *SULT1A1* SNP rs9282862 (p = 7.44 × 10**^–^**^5^) were associated with AUC_0–t_ of total dabigatran. These SNPs also had significant influences on the AUC_0–t_ of free dabigatran, C_max_ and anti-FIIa activity (p < 0.05). 30 new potential SNPs of 13 reported candidate genes (*ABCB1*, *ABCC2*, *ABCG2*, *CYP2B6*, *CYP1A2*, *CYP2C19*, *CYP3A5*, *CES1*, *SLCO1B1*, *SLC22A1*, *UGT1A1*, *UGT1A9*, and *UGT2B7*) that were associated with drug metabolism. |
|  |  |  |  |  |  | *FRAS1* | rs6835769 |  |  |
|  |  |  |  |  |  | *SULT1A1* | rs9282862 |  |  |
|  |  |  |  |  |  | *ABCB1* | rs1045642, rs2235013, rs2235015, rs2235033, rs2235047, rs2235048, rs4148734 |  |  |
|  |  |  |  |  |  | *ABCC2* | rs2273697, rs3740066, rs3740073, rs4148395, rs717620 |  |  |
|  |  |  |  |  |  | *ABCG2* | rs2231138, rs2231142, rs2231148, rs2231156, rs2231157, rs2231165, rs4148152 |  |  |
|  |  |  |  |  |  | *CES1* | rs112236246, rs2244613, rs2244614, rs2302719, rs3217164, rs3815583, rs56278207 |  |  |
|  |  |  |  |  |  | *CYP2C19* | rs12769205, rs17885098, rs3758580, rs4244285 |  |  |
|  |  |  |  |  |  | *CYP2D6* | rs1065852, rs1080995, rs1081003, rs1135840, rs16947, rs28371725 |  |  |
|  |  |  |  |  |  | *SLCO1B1* | rs2291075, rs2291076, rs2306283, rs4149032, rs4149033, rs4149034, rs4149056, rs4149057, rs11045748 |  |  |
|  |  |  |  |  |  | *UGT2B7* | chr4:69879878, rs115791839, rs12233719, rs28365063, rs4257713, rs5013211, rs7438284, rs7658752 |  |  |
|  |  |  |  |  |  | *CYP1A2* | rs4646427 |  |  |
|  |  |  |  |  |  | *CYP2A6* | rs1137115, rs8192720, rs8192725, rs8192726 |  |  |
|  |  |  |  |  |  | *CYP2B6* | rs2279342, rs8192719, rs34433978, rs35930845, rs3745275, rs3745276, rs3745277, rs434606, rs56156262, rs7249735 |  |  |
|  |  |  |  |  |  | *CYP2C8* | rs1058932, rs11572078, rs2071426, rs2275622 |  |  |
|  |  |  |  |  |  | *CYP2J2* | rs2229189, rs2271800 |  |  |
|  |  |  |  |  |  | *CYP3A5* | rs15524, rs4646453 |  |  |
|  |  |  |  |  |  | *CYP4F2* | rs2074900, rs3093106, rs3093160 |  |  |
|  |  |  |  |  |  | *SLC22A1* | rs1867351, rs2282143, rs35854239, rs4646273, rs622591, rs628031, rs683369 |  |  |
|  |  |  |  |  |  | *UGT1A1* | rs4148323, rs2302538, rs4148327 |  |  |
|  |  |  |  |  |  | *UGT1A9* | rs12466997, rs2361501, rs869283, rs7563561, rs7608175, rs7586110 |  |  |
| **II. AF Patients** | | | | | | | | | |
| Paré G et al, 2013 [5]^b,e^ | 1694 | 71.8±7.5^a^ | 1163(68.7) | NA | European Caucasian | *ABCB1* | rs4148738, rs2235046, rs1128503, rs10276036, rs1202169, rs1202168, rs1202167, rs8192935 | C_trough_, C_peak_, bioavailability, volume of distribution, clearance, ischemic events, bleeding events | Each minor allele (C) of *CES1* rs2244613 was associated with lower C_trough_ (p=1.2×10**^–^**^8^) and a lower risk of any bleeding (p=7×10**^–^**^8^), and *ABCB1* rs4148738 and *CES1* rs8192935 were associated with C_trough_ at genome-wide significance (p < 9×10**^–^**^8^) with a gene-dose effect. |
|  |  |  |  |  |  | *CES1* | rs2244613, rs4122238, rs8192935 |  |  |
|  |  |  |  |  |  | *CES1P2* | rs4580160, rs4784563 |  |  |
| Chin PK et al, 2014 [6]^b^ | 52 | 67.0±14.0^a^ | 41(78.8) | 95.0±32.8^a^ | Caucasian | *ABCB1* | rs1045642, rs1128503, rs4148738, rs2032582 | PDC | No significant effect. |
|  |  |  |  |  |  | *CES1* | rs2244613, rs8192935, rs412223 |  |  |
| Tomita H et al, 2016 [7]^b^ | 98 | 71.4±8.4 | 64(65.3) | 62.0±12.6 | Japanese | *ABCB1* | rs1045642, rs2032582, rs1128503 | C_trough_, 90min PDC, APTT, DD | No significant effect. Besides, this study only investigated the association between *ABCB1* and PDC, no with APTT and DD. |
| Dimatteo C et al, 2016 [8] | 92 | 72.0±6.7^a^ | 51(55.4) | NA | Caucasian | *ABCB1* | rs4148738 | C_trough_, C_peak_ | The *CES1* rs8192935 significantly influenced the dabigatran trough concentrations (p = 0.023) and carriers of the T allele showed significantly lower concentrations than did carriers of the CC genotype. |
|  |  |  |  |  |  | *CES1* | rs2244613, rs8192935 |  |  |
| Sychev D et al, 2020 [9]^c^ | 96 | 75.0±6.3^a^ | 39(40.6) | NA | Russian | *ABCB1* | rs1045642, rs4148738 | C/D ratio, bleeding | Patients with the rs2244613 *CC* genotype had lower C/D values (70% reduction in the mean C/D vs. *AA* genotype, p = 0.001). Low number of bleeding events precluded any analyses between genotypes and clinical outcomes. |
|  |  |  |  |  |  | *CES1* | rs2244613 |  |  |
| Roşian AN et al, 2020 [10] | 104 | 70.89±8.85 | 55(52.88) | NA | Caucasian | *ABCB1* | rs1045642, rs4148738 | Non-major bleeding | No significant associations. A trend of association between TG haplotype with bleeding risk was observed. |
| Ji Q et al, 2021 [11]^b^ | 198 | 63.3±9.3 | 120(60.6) | NA | Chinese | *ABCB1*  *CES1* | rs1045642, rs4148738 | PDC, APTT, TT, bleeding | The minor allele (C) on the *CES1* rs8192935 was associated with PDCs and APTT values at trough level (P = .028 for peak PDC, P < 0.001 for trough PDC, P = .015 for APTT). The minor allele (A) on the *CES1* rs2244613 was associated with increased trough PDCs (ANOVA: P < .001; AA vs. CC, P < .001; CA vs. CC, P = 0.004) and higher risk for minor bleeding (p = 0.034). |
|  |  |  |  |  |  |  | rs2244613, rs8192935 |  |  |
| Lähteenmäki J et al, 2021 [12]^d^ | 340 | 69.8±8.7 | 178(52.4) | NA | Finnish | *ABCB1* | rs1045642, rs2032582, rs4148738, rs1128503 | Bleeding and thromboembolic events | No significant effect. |
|  |  |  |  |  |  | *ABCG2* | rs2231142 |  |  |
|  |  |  |  |  |  | *CES1* | rs2244613, rs8192935 |  |  |
|  |  |  |  |  |  | *CYP3A5* | rs776746 |  |  |

PK, pharmacokinetic; C_trough_, trough plasma concentration, 10–16 h after previous dose; C_peak_, peak plasma concentration, 1–3 h after the previous dose; C_max_, maximum plasma concentration; AUC_0–24h_, area under the curve from the time of dosing to 24 h after dosing; AUC_last_, area under the curve from the time of dosing to the last measurable concentration; AUC_0-∞_, area under the curve from the time of dosing to the last measurable concentration and extrapolated to infinity; T_max_, time to peak concentration; C/D ratio, trough plasma concentration/dose ratio; t_½_, half-life; CL/F, apparent oral clearance; Vd/F, apparent volume of distribution; AUC_0–12h_, area under the curve from the time of dosing to 12 h after dosing; AUC/DW, variables AUC_∞_ were divided by the dose/weight(DW) ratio; C_max_, variables C_max_ were divided by the DW; APTT, activated partial thromboplastin time; PT, prothrombin time; PDC, plasma dabigatran concentration; DD, D-dimer; TT, thrombin time; AF, atrial fibrillation

Data of age and weight were shown as “mean ± SD”

a: calculated according to the data of text

b: The category of AF was nonvalvular AF

c: AF patients with chronic kidney disease stage 3A-3B

d: Indication for dabigatran including: AF (78.8%); vascular disease (9.4%); pulmonary embolism (2.9%); stroke, cerebral infarction, atherosclerosis (4.7%); venous thrombosis (4.1%)

e: Only this study method was genome-wide association analysis. All the others’ methods were candidate gene analysis.

**Table S2. Detailed clinical outcomes of patients treated with dabigatran at all follow-up visits**

| **Events** | | **0-1 month** | | **2-6 months** | | **7-12 months** | | **13-24 months** | |
| --- | --- | --- | --- | --- | --- | --- | --- | --- | --- |
|  |  | **n** | **details** | **n** | **details** | **n** | **details** | **n** | **details** |
| **Bleeding^#^** | BARC type 1 | 7 | gingival (4)  subcutaneous (1)  gastrointestinal (1)  pharyngeal (1) | 10 | gingival (4)  subcutaneous (2)  nasal (2)  conjunctival (1)  gastrointestinal (1)  hematuria (1) | 10 | gingival (4)  subcutaneous (3)  nasal (1)  gastrointestinal (2)  microscopic hematuria (1) | 3 | gingival (1)  microscopic hematuria (1)  hematuria (1) |
|  | BARC type 2 | 0 |  | 1 | gingival and nasal (1) | 0 |  | 0 |  |
|  | BARC type 3b | 0 |  | 0 |  | 1 | hematuria intervened by surgery (1) | 0 |  |
|  | BARC type 3c | 0 |  | 0 |  | 1 | intracranial hemorrhage (1) | 0 |  |
| **TE** | MI | 0 |  | 0 |  | 1 |  | 0 |  |
|  | Stroke | 0 |  | 2 | ischemic stroke (2) | 2 | hemorrhagic stroke (2, one was microbleed) | 2 | ischemic stroke (2) |
|  | SE | 2 |  | 0 |  | 0 |  | 0 |  |
| **MACE** | MI | 0 |  | 0 |  | 1 |  | 0 |  |
|  | Stroke | 0 |  | 2 | ischemic stroke (2) | 2 | hemorrhagic stroke (2, one was microbleed) | 2 | ischemic stroke (2) |
|  | Repeated revascularization | 0 |  | 0 |  | 1 |  | 0 |  |

TE, thromboembolic events; MACE, major adverse cardiac event; MI, myocardial infarction; SE, systemic embolism.

^#^Some patients suffered more than 2 types of bleeding

**Table S3. Effects of *UBASH3B* rs2276408 and *FBN2* rs3805625 on the pharmacodynamics of dabigatran**

| **Gene** | **SNP** | **Genotypes#** | **Peak PD levels** | | | | | | | **Trough PD levels** | | | | | | |
| --- | --- | --- | --- | --- | --- | --- | --- | --- | --- | --- | --- | --- | --- | --- | --- | --- |
|  |  |  | **GENO*** | **FIIa(ng/ml)** | **p-value** | **FAPTT(s)** | **p-value** | **FPT(s)** | **p-value** | **GENO** | **GIIa(ng/ml)** | **p-value** | **GAPTT(s)** | **p-value** | **GPT(s)** | **p-value** |
| *UBASH3B* | rs2276408 | A1A1 | 2/29/124 | 330.93±44.08 | **0.024** | 56.35±4.35 | 0.083 | 16.55±1.05 | 0.106 | 2/26/123 | 80.15±2.35 | 0.092 | 39.65±2.55 | 0.819 | 12.90±0.90 | 0.063 |
|  |  | A1A2 |  | 194.21±145.66 |  | 52.20±15.44 |  | 14.53±2.44 |  |  | 88.06±69.49 |  | 39.64±8.93 |  | 12.77±1.33 |  |
|  |  | A2A2 |  | 137.61±119.31 |  | 47.24±13.13 |  | 13.94±3.82 |  |  | 63.75±57.56 |  | 39.53±10.13 |  | 13.94±6.70 |  |
| *FBN2* | rs3805625 | A1A1 | 3/28/124 | 114.14±76.66 | 0.329 | 38.03±12.78 | 0.840 | 12.37±1.81 | 0.585 | 3/27/121 | 16.74±20.53 | 0.505 | 33.38±4.02 | 0.189 | 13.90±3.18 | 0.694 |
|  |  | A1A2 |  | 204.50±128.11 |  | 50.03±11.09 |  | 14.33±2.24 |  |  | 85.69±63.09 |  | 38.31±7.88 |  | 12.65±1.47 |  |
|  |  | A2A2 |  | 139.41±125.27 |  | 48.08±14.11 |  | 14.08±3.86 |  |  | 65.52±58.93 |  | 39.98±10.29 |  | 13.96±6.72 |  |

SNP, single nucleotide polymorphism; GENO, Number of each genotype (A1A1/A1A2/A2A2); A1, minor allele; A2, non- minor allele; *UBASH3B*, ubiquitin associated and SH3 domain containing B; *FBN2*, fibrillin 2.

# *UBASH3B* SNP rs2276408: A1= T, A2= C; *FBN2* SNP rs3805625: A1= T, A2= G

*As the number of peak anti-FIIa activity was 156 and the number of peak APTT and PT was 155, the genotype of extra one which had only peak anti-FIIa activity was: *UBASH3B* SNP rs2276408: A2A2; *FBN2* SNP rs3805625.

**Table S4. Effects of suggestive SNPs associated with PD parameters on bleeding events of patients treated with dabigatran**

| **Gene** | **SNP** | **Bleeding events, n(%)** | **GENO** | **A1A1^#^** | **A1A2^#^** | **A2A2^#^** | **OR(95%CI)** | **p value** |
| --- | --- | --- | --- | --- | --- | --- | --- | --- |
| *SNX7* | rs9433747 | 1 month | 0/28/141 | / | 2(7.1) | 5(3.5) | 2.04(0.37-11.19) | 0.410 |
|  |  | 6 months | 0/28/121 | / | 4(14.3) | 12(9.9) | 1.49(0.44-5.10) | 0.524 |
|  |  | 12 months | 0/26/96 | / | 8(30.8) | 16(16.7) | 2.29(0.83-6.31) | 0.109 |
|  |  | 24 months | 0/13/50 | / | 9(69.2) | 17(34.0) | 4.76(1.20-18.95) | **0.027** |
| *BRD4* | rs11669901 | 1 month | 1/18/150 | 0(0.00) | 2(11.1) | 5(3.3) | 2.85(0.58-13.91) | 0.195 |
|  |  | 6 months | 1/17/131 | 0(0.00) | 3(17.6) | 13(9.9) | 1.46(0.40-5.37) | 0.572 |
|  |  | 12 months | 1/15/106 | 0(0.00) | 7(46.7) | 17(16.0) | 2.87(1.00-8.28) | 0.051 |
|  |  | 24 months | 0/10/53 | / | 7(70.0) | 19(35.8) | 3.99(0.89-17.84) | 0.070 |
| *FLCN* | rs3744124 | 1 month | 6/58/105 | 0(0.00) | 3(5.2) | 4(3.8) | 1.20(0.29-5.01) | 0.799 |
|  |  | 6 months | 5/53/91 | 1(20.0) | 4(7.5) | 11(12.1) | 1.02(0.38-2.77) | 0.966 |
|  |  | 12 months | 5/40/77 | 1(20.0) | 7(17.5) | 16(20.8) | 0.91(0.39-2.11) | 0.818 |
|  |  | 24 months | 4/16/43 | 1(25.0) | 7(43.8) | 18(41.9) | 0.73(0.28-1.94) | 0.532 |
| *UBAP1* | rs1556439 | 1 month | 1/16/152 | 0(0.00) | 2(12.5) | 5(3.3) | 3.07(0.63-14.94) | 0.166 |
|  |  | 6 months | 1/14/134 | 1(100.0) | 2(14.3) | 13(9.7) | 2.45(0.74-8.07) | 0.141 |
|  |  | 12 months | 1/12/109 | 1(100.0) | 2(16.7) | 21(19.3) | 1.48(0.45-4.81) | 0.518 |
|  |  | 24 months | 1/8/54 | 1(100.0) | 3(37.5) | 22(40.7) | 1.47(0.41-5.31) | 0.557 |
| *IGLV3-12* | rs2073451 | 1 month | 35/78/56 | 1(2.9) | 3(3.8) | 3(5.4) | 0.70(0.23-2.12) | 0.531 |
|  |  | 6 months | 30/68/51 | 4(13.3) | 8(11.8) | 4(7.8) | 1.43(0.69-2.94) | 0.334 |
|  |  | 12 months | 22/56/44 | 5(22.7) | 12(21.4) | 7(15.9) | 1.35(0.71-2.56) | 0.353 |
|  |  | 24 months | 10/29/24 | 5(50.0) | 12(41.4) | 9(37.5) | 1.53(0.71-3.28) | 0.274 |
| *LRRC8E* | rs3745382 | 1 month | 4/31/134 | 0(0.00) | 2(6.5) | 5(3.7) | 1.21(0.28-5.20) | 0.794 |
|  |  | 6 months | 3/28/118 | 0(0.00) | 2(7.1) | 14(11.9) | 0.50(0.12-2.12) | 0.346 |
|  |  | 12 months | 3/21/98 | 1(33.3) | 2(9.5) | 21(21.4) | 0.68(0.23-2.00) | 0.480 |
|  |  | 24 months | 2/9/52 | 1(50.0) | 2(22.2) | 23(44.2) | 0.62(0.19-2.02) | 0.426 |
| *PTPLAD1* | rs11539008 | 1 month | 0/25/144 | / | 0(0.00) | 7(0.49) | / | / |
|  |  | 6 months | 0/21/128 | / | 0(0.00) | 16(12.5) | / | / |
|  |  | 12 months | 0/16/106 | / | 2(12.5) | 22(20.8) | 0.55(0.12-2.66) | 0.460 |
|  |  | 24 months | 0/10/53 | / | 3(30.0) | 23(43.4) | 0.55(0.12-2.41) | 0.426 |
| *ZNF230* | rs12753 | 1 month | 12/59/99 | 0(0.00) | 4(6.9) | 3(3.0) | 1.23(0.39-3.84) | 0.726 |
|  |  | 6 months | 10/52/87 | 0(0.00) | 7(13.5) | 9(10.3) | 0.84(0.35-2.04) | 0.701 |
|  |  | 12 months | 8/44/70 | 1(12.5) | 8(18.2) | 15(21.4) | 0.78(0.36-1.71) | 0.539 |
|  |  | 24 months | 2/23/38 | 1(50.0) | 9(39.1) | 16(42.1) | 0.90(0.35-2.27) | 0.817 |
| *ANP32A* | rs12904108 | 1 month | 6/47/116 | 0(0.00) | 1(2.1) | 6(5.2) | 0.34(0.04-2.66) | 0.303 |
|  |  | 6 months | 6/43/100 | 0(0.00) | 3(7.0) | 13(13.0) | 0.43(0.12-1.48) | 0.180 |
|  |  | 12 months | 5/34/83 | 0(0.00) | 6(17.6) | 18(21.7) | 0.86(0.25-1.52) | 0.288 |
|  |  | 24 months | 1/18/44 | 0(0.00) | 8(44.4) | 18(40.9) | 0.86(0.31-2.40) | 0.768 |
| *SLC25A28* | rs12252561 | 1 month | 9/48/112 | 1(11.1) | 2(4.2) | 4(3.6) | 1.55(0.48-5.05) | 0.463 |
|  |  | 6 months | 9/40/100 | 1(11.1) | 3(7.5) | 12(12.0) | 0.65(0.24-1.77) | 0.397 |
|  |  | 12 months | 8/34/80 | 2(25.0) | 5(14.7) | 17(21.3) | 0.86(0.40-1.85) | 0.691 |
|  |  | 24 months | 4/18/41 | 2(50.0) | 7(38.9) | 17(41.5) | 0.99(0.43-2.30) | 0.980 |
| *ABCC2* | rs2273697 | 1 month | 3/24/142 | 0(0.00) | 0(0.00) | 7(4.9) | / | / |
|  |  | 6 months | 3/19/127 | 0(0.00) | 1(5.3) | 15(11.8) | 0.31(0.04-2.28) | 0.249 |
|  |  | 12 months | 3/16/103 | 1(33.3) | 2(12.5) | 21(20.4) | 0.81(0.28-2.34) | 0.701 |
|  |  | 24 months | 2/9/52 | 1(50.0) | 4(44.4) | 21(40.4) | 1.01(0.34-2.97) | 0.990 |
| *ABCC2* | rs4148395 | 1 month | 3/24/142 | 0(0.00) | 0(0.00) | 7(4.9) | / | / |
|  |  | 6 months | 3/19/127 | 0(0.00) | 1(5.3) | 15(11.8) | 0.31(0.04-2.28) | 0.249 |
|  |  | 12 months | 3/16/103 | 1(33.3) | 2(12.5) | 21(20.4) | 0.81(0.28-2.34) | 0.701 |
|  |  | 24 months | 2/9/52 | 1(50.0) | 4(44.4) | 21(40.4) | 1.01(0.34-2.97) | 0.990 |
| *MYBPC1* | rs11110942 | 1 month | 7/65/97 | 0(0.00) | 4(6.2) | 3(3.1) | 1.33(0.38-4.70) | 0.657 |
|  |  | 6 months | 7/58/84 | 0(0.00) | 9(15.5) | 7(8.3) | 1.28(0.55-2.99) | 0.566 |
|  |  | 12 months | 7/45/70 | 0(0.00) | 11(24.4) | 13(18.6) | 0.94(0.44-1.98) | 0.864 |
|  |  | 24 months | 2/24/37 | 1(50.0) | 11(45.8) | 14(37.8) | 1.30(0.52-3.24) | 0.577 |
| *MYBPC1* | rs3751246 | 1 month | 7/66/97 | 0(0.00) | 4(6.1) | 3(3.1) | 1.31(0.37-4.65) | 0.673 |
|  |  | 6 months | 7/59/83 | 0(0.00) | 10(16.9) | 6(7.2) | 1.50(0.65-3.45) | 0.346 |
|  |  | 12 months | 7/46/69 | 0(0.00) | 12(26.1) | 12(17.4) | 1.04(0.50-2.17) | 0.920 |
|  |  | 24 months | 2/25/36 | 1(50.0) | 12(48.0) | 13(36.1) | 1.46(0.58-3.65) | 0.418 |
| *MYBPC1* | rs11110952 | 1 month | 7/67/95 | 0(0.00) | 4(6.0) | 3(3.2) | 1.29(0.36-4.59) | 0.697 |
|  |  | 6 months | 7/60/82 | 0(0.00) | 10(16.7) | 6(7.3) | 1.48(0.64-3.41) | 0.363 |
|  |  | 12 months | 7/47/68 | 0(0.00) | 12(25.5) | 12(17.6) | 1.02(0.49-2.13) | 0.962 |
|  |  | 24 months | 2/25/36 | 1(50.0) | 12(48.0) | 13(36.1) | 1.46(0.58-3.65) | 0.418 |
| *CEP170B* | rs60001925 | 1 month | 3/37/129 | 1(33.3) | 1(2.7) | 5(3.9) | 2.01(0.53-7.65) | 0.304 |
|  |  | 6 months | 3/35/111 | 1(33.3) | 4(11.4) | 11(9.9) | 1.47(0.56-3.86) | 0.439 |
|  |  | 12 months | 3/29/90 | 1(33.3) | 4(13.8) | 19(21.1) | 0.85(0.34-2.16) | 0.738 |
|  |  | 24 months | 1/14/48 | 1(100.0) | 5(35.7) | 20(41.7) | 1.31(0.43-4.04) | 0.633 |
| *GYPA* | rs145195209 | 1 month | 2/28/139 | 0(0.00) | 0(0.00) | 7(5.0) | / | / |
|  |  | 6 months | 2/23/124 | 0(0.00) | 2(8.7) | 14(11.3) | 0.67(0.16-2.77) | 0.577 |
|  |  | 12 months | 1/16/105 | 0(0.00) | 3(18.8) | 21(20.0) | 0.79(0.23-2.78) | 0.718 |
|  |  | 24 months | 0/7/56 | / | 3(42.9) | 23(41.1) | 1.12(0.22-5.70) | 0.896 |

SNP, single nucleotide polymorphism; CHR, chromosome; Func, the region of the genome where the mutation is; GENO, Number of each genotype (A1A1/A1A2/A2A2); A1, minor allele; A2, non- minor allele; OR, odds ratio; CI, confidence interval.

# *SNX7* SNP rs9433747: A1=G, A2=A; *BRD4* SNP rs11669901: A1=A, A2=G; *FLCN* SNP rs3744124: A1=T, A2=C; *UBAP1* SNP rs1556439: A1=T, A2=C; *IGLV3-12* SNP rs2073451: A1=G, A2=A; *LRRC8E* SNP rs3745382: A1=A, A2=G; *PTPLAD1* SNP rs11539008: A1=A, A2=G; *ZNF230* SNP rs12753: A1=A, A2=C; *ANP32A* SNP rs12904108: A1=T, A2=A; *SLC25A28* SNP rs12252561: A1=C, A2=G; *ABCC2* SNP rs2273697: A1=A, A2=G; *ABCC2* SNP rs4148395: A1=A, A2=G; *MYBPC1* SNP rs11110942: A1=G, A2=C; *MYBPC1* SNP rs3751246: A1=T, A2=C; *MYBPC1* SNP rs11110952: A1=C, A2=T; *CEP170B* SNP rs60001925: A1=C, A2=CGCAGGA; *GYPA* SNP rs145195209: A1=A, A2=AT.

**Table S5. Negative effects of candidate genes on bleeding and the pharmacodynamics of dabigatran**

| **SNP** | **Gene** | **Bleeding**  **1 month** | **Bleeding**  **6 months** | **Bleeding**  **12 months** | **Bleeding**  **24 months** | **FIIa**  **(ng/ml)** | **FAPTT**  **(s)** | **FPT**  **(s)** | **GIIa**  **(ng/ml)** | **GAPTT**  **(s)** | **GPT**  **(s)** |
| --- | --- | --- | --- | --- | --- | --- | --- | --- | --- | --- | --- |
|  |  | **p-values** | | | | | | | | | |
| rs1045642#* | *ABCB1* | 0.420 | 0.750 | 0.821 | 0.479 | 0.751 | 0.259 | 0.971 | 0.083 | 0.247 | 0.793 |
| rs2235047* | *ABCB1* | 0.928 | 0.671 | 0.571 | 0.458 | 0.459 | 0.268 | 0.529 | 0.090 | 0.400 | 0.873 |
| rs2235048* | *ABCB1* | 0.436 | 0.694 | 0.750 | 0.479 | 0.741 | 0.247 | 0.861 | 0.098 | 0.256 | 0.786 |
| rs4148734* | *ABCB1* | 0.279 | 0.959 | 0.264 | 0.072 | 0.437 | 0.503 | 0.888 | 0.841 | 0.380 | 0.874 |
| rs36014243 | *ABCB1, RUNDC3B* | 0.435 | 0.832 | 0.798 | 0.360 | 0.778 | 0.920 | 0.544 | 0.872 | 0.909 | 0.840 |
| rs3740066* | *ABCC2* | 0.852 | 0.884 | 0.278 | 0.244 | 0.868 | 0.506 | 0.399 | 0.781 | 0.318 | 0.593 |
| rs3740073* | *ABCC2* | 0.861 | 0.899 | 0.298 | 0.244 | 0.868 | 0.506 | 0.399 | 0.808 | 0.241 | 0.593 |
| rs717620* | *ABCC2* | 0.988 | 0.796 | 0.211 | 0.190 | 0.908 | 0.573 | 0.399 | 0.726 | 0.343 | 0.572 |
| rs2231138* | *ABCG2* | 0.738 | 0.250 | 0.300 | 0.651 | 0.759 | 0.653 | 0.798 | 0.867 | 0.169 | 0.623 |
| rs2231157* | *ABCG2* | 0.146 | 0.171 | 0.379 | 0.372 | 0.387 | 0.483 | 0.136 | 0.722 | 0.620 | 0.418 |
| rs4148152* | *ABCG2* | 0.586 | 0.967 | 0.531 | 0.312 | 0.926 | 0.285 | 0.273 | 0.910 | 0.980 | 0.289 |
| rs2244613#* | *CES1* | 0.769 | 0.099 | 0.213 | 0.140 | 0.866 | 0.851 | 0.504 | 0.687 | 0.150 | 0.092 |
| rs2302719* | *CES1* | 0.911 | 0.142 | 0.278 | 0.208 | 0.907 | 0.778 | 0.497 | 0.996 | 0.093 | 0.089 |
| rs2307240 | *CES1* | 0.239 | 0.978 | 0.546 | 0.978 | 0.819 | 0.989 | 0.573 | 0.787 | 0.737 | 0.719 |
| rs3815583* | *CES1* | 0.964 | 0.794 | 0.985 | 0.929 | 0.900 | 0.331 | 0.506 | 0.643 | 0.782 | 0.228 |
| rs3848300 | *CES1* | 0.194 | 0.062 | 0.301 | 0.225 | 0.884 | 0.829 | 0.240 | 0.842 | 0.220 | 0.069 |
| rs56278207* | *CES1* | 0.633 | 0.117 | 0.147 | 0.238 | 0.571 | 0.754 | 0.680 | 0.599 | 0.654 | 0.756 |
| rs1137115* | *CYP2A6* | 0.572 | 0.587 | 0.699 | 0.613 | 0.596 | 0.769 | 0.806 | 0.756 | 0.577 | 0.612 |
| rs8192725* | *CYP2A6* | 0.572 | 0.587 | 0.699 | 0.613 | 0.596 | 0.769 | 0.806 | 0.756 | 0.577 | 0.612 |
| rs2279342* | *CYP2B6* | 0.796 | 0.924 | 0.851 | 0.551 | 0.421 | 0.952 | 0.746 | 0.679 | 0.091 | 0.429 |
| rs8192709 | *CYP2B6* | 0.765 | 0.365 | 0.333 | 0.825 | 0.718 | 0.480 | 0.108 | 0.507 | 0.923 | 0.711 |
| rs35930845* | *CYP2B6, CYP2A13* | 0.224 | 0.964 | 0.460 | 0.544 | 0.401 | 0.296 | 0.191 | 0.141 | 0.487 | 0.850 |
| rs434606* | *CYP2B6, CYP2A13* | 0.793 | 0.489 | 0.283 | 0.100 | 0.084 | 0.875 | 0.932 | 0.128 | 0.106 | 0.635 |
| rs56156262* | *CYP2B6, CYP2A13* | 0.162 | 0.965 | 0.784 | 0.643 | 0.374 | 0.914 | 0.650 | 0.151 | 0.559 | 0.842 |
| rs12769205* | *CYP2C19* | 0.361 | 0.713 | 0.892 | 0.589 | 0.358 | 0.916 | 0.467 | 0.054 | 0.438 | 0.100 |
| rs17885098* | *CYP2C19* | 0.097 | 0.769 | 0.723 | 0.772 | 0.720 | 0.555 | 0.871 | 0.210 | 0.090 | 0.897 |
| rs3758580* | *CYP2C19* | 0.361 | 0.713 | 0.892 | 0.589 | 0.358 | 0.916 | 0.467 | 0.054 | 0.438 | 0.100 |
| rs4244285** | *CYP2C19* | 0.361 | 0.713 | 0.892 | 0.589 | 0.358 | 0.916 | 0.467 | 0.054 | 0.438 | 0.100 |
| rs2071426* | *CYP2C8* | 0.366 | 0.235 | 0.382 | 0.392 | 0.131 | 0.595 | 0.585 | 0.514 | 0.842 | 0.771 |
| rs1065852** | *CYP2D6* | 0.577 | 0.494 | 0.741 | 0.789 | 0.947 | 0.531 | 0.631 | 0.673 | 0.221 | 0.510 |
| rs1080995* | *CYP2D6* | 0.722 | 0.694 | 0.849 | 0.373 | 0.743 | 0.737 | 0.929 | 0.550 | 0.839 | 0.931 |
| rs1080996 | *CYP2D6* | 0.722 | 0.694 | 0.849 | 0.373 | 0.743 | 0.737 | 0.929 | 0.550 | 0.839 | 0.931 |
| rs1081003* | *CYP2D6* | 0.555 | 0.521 | 0.553 | 0.989 | 0.826 | 0.549 | 0.670 | 0.422 | 0.306 | 0.605 |
| rs1135840* | *CYP2D6* | 0.692 | 0.641 | 0.614 | 0.735 | 0.770 | 0.733 | 0.551 | 0.971 | 0.210 | 0.432 |
| rs16947* | *CYP2D6* | 0.745 | 0.668 | 0.894 | 0.480 | 0.684 | 0.656 | 0.978 | 0.529 | 0.842 | 0.967 |
| rs2229189* | *CYP2J2* | / | / | / | / | 0.127 | 0.321 | 0.497 | 0.070 | 0.588 | 0.573 |
| rs2271800* | *CYP2J2* | 0.565 | 0.777 | 0.693 | 0.915 | 0.681 | 0.818 | 0.560 | 0.357 | 0.725 | 0.083 |
| rs15524* | *CYP3A5* | 0.805 | 0.313 | 0.211 | 0.454 | 0.564 | 0.913 | 0.923 | 0.391 | 0.136 | 0.484 |
| rs4646453* | *CYP3A5, ZSCAN25* | 0.908 | 0.191 | 0.516 | 0.891 | 0.542 | 0.926 | 0.836 | 0.940 | 0.303 | 0.374 |
| rs2074900* | *CYP4F2* | 0.497 | 0.919 | 0.681 | 0.542 | 0.679 | 0.082 | 0.939 | 0.956 | 0.702 | 0.972 |
| rs3093106* | *CYP4F2* | 0.999 | 0.539 | 0.293 | 0.570 | 0.271 | 0.566 | 0.143 | 0.056 | 0.469 | 0.923 |
| rs3093160* | *CYP4F2* | 0.999 | 0.539 | 0.293 | 0.570 | 0.271 | 0.566 | 0.143 | 0.056 | 0.469 | 0.923 |
| rs10008489 | *FRAS1* | 0.806 | 0.347 | 0.568 | 0.627 | 0.215 | 0.822 | 0.802 | 0.298 | 0.444 | 0.886 |
| rs10010238 | *FRAS1* | 0.799 | 0.763 | 0.120 | 0.247 | 0.263 | 0.776 | 0.810 | 0.971 | 0.494 | 0.206 |
| rs10016229 | *FRAS1* | 0.806 | 0.347 | 0.568 | 0.627 | 0.215 | 0.822 | 0.802 | 0.298 | 0.444 | 0.886 |
| rs10018235 | *FRAS1* | 0.414 | 0.665 | 0.931 | 0.709 | 0.402 | 0.879 | 0.827 | 0.521 | 0.353 | 0.723 |
| rs10026662 | *FRAS1* | 0.413 | 0.668 | 0.868 | 0.709 | 0.313 | 0.994 | 0.965 | 0.449 | 0.353 | 0.728 |
| rs1017646 | *FRAS1* | 0.947 | 0.904 | 0.101 | 0.333 | 0.431 | 0.936 | 0.737 | 0.934 | 0.729 | 0.301 |
| rs1017647 | *FRAS1* | 0.947 | 0.904 | 0.101 | 0.333 | 0.431 | 0.936 | 0.737 | 0.934 | 0.729 | 0.301 |
| rs1017648 | *FRAS1* | 0.947 | 0.904 | 0.101 | 0.333 | 0.431 | 0.936 | 0.737 | 0.934 | 0.729 | 0.301 |
| rs12512164 | *FRAS1* | 0.949 | 0.548 | 0.641 | 0.262 | 0.772 | 0.302 | 0.153 | 0.068 | 0.196 | 0.115 |
| rs13117388 | *FRAS1* | 0.704 | 0.324 | 0.355 | 0.518 | 0.760 | 0.679 | 0.954 | 0.966 | 0.763 | 0.406 |
| rs1471752 | *FRAS1* | 0.410 | 0.810 | 0.631 | 0.294 | 0.659 | 0.141 | 0.227 | 0.199 | 0.111 | 0.954 |
| rs1496598 | *FRAS1* | 0.757 | 0.523 | 0.889 | 0.278 | 0.502 | 0.711 | 0.947 | 0.538 | 0.335 | 0.625 |
| rs2280223 | *FRAS1* | 0.142 | 0.815 | 0.990 | 0.732 | 0.510 | 0.567 | 0.143 | 0.086 | 0.123 | 0.379 |
| rs28566963 | *FRAS1* | 0.414 | 0.665 | 0.931 | 0.709 | 0.402 | 0.879 | 0.827 | 0.521 | 0.353 | 0.723 |
| rs2867014 | *FRAS1* | 0.947 | 0.904 | 0.101 | 0.333 | 0.431 | 0.936 | 0.737 | 0.934 | 0.729 | 0.301 |
| rs34015316 | *FRAS1* | 0.664 | 0.355 | 0.872 | 0.215 | 0.837 | 0.711 | 0.779 | 0.922 | 0.941 | 0.122 |
| rs345513 | *FRAS1* | 0.727 | 0.126 | 0.769 | 0.583 | 0.861 | 0.409 | 0.942 | 0.893 | 0.687 | 0.057 |
| rs345514 | *FRAS1* | 0.281 | 0.741 | 0.842 | 0.354 | 0.484 | 0.932 | 0.223 | 0.372 | 0.236 | 0.891 |
| rs345528 | *FRAS1* | 0.947 | 0.904 | 0.101 | 0.333 | 0.431 | 0.936 | 0.737 | 0.934 | 0.729 | 0.301 |
| rs34670941 | *FRAS1* | 0.225 | 0.117 | 0.123 | 0.469 | 0.444 | 0.690 | 0.689 | 0.892 | 0.724 | 0.612 |
| rs34840208 | *FRAS1* | 0.422 | 0.662 | 0.867 | 0.709 | 0.354 | 0.861 | 0.871 | 0.422 | 0.288 | 0.701 |
| rs34880017 | *FRAS1* | 0.449 | 0.750 | 0.692 | 0.294 | 0.660 | 0.141 | 0.176 | 0.236 | 0.072 | 0.935 |
| rs3749487 | *FRAS1* | 0.219 | 0.104 | 0.101 | 0.339 | 0.444 | 0.690 | 0.689 | 0.915 | 0.745 | 0.649 |
| rs3749488 | *FRAS1* | 0.815 | 0.466 | 0.815 | 0.364 | 0.503 | 0.640 | 0.995 | 0.491 | 0.293 | 0.606 |
| rs3749489 | *FRAS1* | 0.075 | 0.499 | 0.277 | 0.161 | 0.163 | 0.613 | 0.653 | 0.402 | 0.424 | 0.972 |
| rs4859905 | *FRAS1* | 0.771 | 0.042 | 0.410 | 0.269 | 0.791 | 0.267 | 0.372 | 0.393 | 0.337 | 0.974 |
| rs4975070 | *FRAS1* | 0.046 | 0.205 | 0.154 | 0.200 | 0.492 | 0.539 | 0.798 | 0.851 | 0.637 | 0.163 |
| rs4975139 | *FRAS1* | 0.046 | 0.205 | 0.154 | 0.200 | 0.492 | 0.539 | 0.798 | 0.851 | 0.637 | 0.163 |
| rs6835769* | *FRAS1* | 0.844 | 0.305 | 0.422 | 0.719 | 0.856 | 0.740 | 0.399 | 0.242 | 0.132 | 0.586 |
| rs72866323 | *FRAS1* | 0.954 | 0.886 | 0.099 | 0.226 | 0.484 | 0.577 | 0.423 | 0.969 | 0.560 | 0.268 |
| rs753752 | *FRAS1* | 0.413 | 0.668 | 0.868 | 0.709 | 0.313 | 0.994 | 0.965 | 0.449 | 0.353 | 0.728 |
| rs7660664 | *FRAS1* | 0.230 | 0.155 | 0.126 | 0.148 | 0.550 | 0.642 | 0.688 | 0.976 | 0.664 | 0.626 |
| rs7694740 | *FRAS1* | 0.673 | 0.369 | 0.872 | 0.215 | 0.837 | 0.711 | 0.779 | 0.922 | 0.941 | 0.122 |
| rs78711748 | *FRAS1* | 0.953 | 0.755 | 0.101 | 0.333 | 0.142 | 0.656 | 0.879 | 0.711 | 0.838 | 0.401 |
| rs931606 | *FRAS1* | 0.757 | 0.523 | 0.889 | 0.278 | 0.502 | 0.711 | 0.947 | 0.538 | 0.335 | 0.625 |
| rs1867351* | *SLC22A1* | 0.664 | 0.930 | 0.102 | 0.261 | 0.453 | 0.926 | 0.268 | 0.646 | 0.369 | 0.231 |
| rs2282143* | *SLC22A1* | 0.470 | 0.796 | 0.565 | 0.408 | 0.322 | 0.872 | 0.519 | 0.304 | 0.427 | 0.747 |
| rs35854239* | *SLC22A1* | 0.523 | 0.269 | 0.057 | 0.236 | 0.449 | 0.650 | 0.954 | 0.458 | 0.691 | 0.678 |
| rs4646273* | *SLC22A1* | 0.680 | 0.922 | 0.104 | 0.261 | 0.453 | 0.926 | 0.268 | 0.748 | 0.294 | 0.231 |
| rs622591* | *SLC22A1* | 0.318 | 0.218 | 0.696 | 0.435 | 0.608 | 0.784 | 0.697 | 0.540 | 0.125 | 0.374 |
| rs628031* | *SLC22A1* | 0.523 | 0.269 | 0.057 | 0.236 | 0.449 | 0.650 | 0.954 | 0.458 | 0.691 | 0.678 |
| rs683369* | *SLC22A1* | 0.405 | 0.947 | 0.661 | 0.262 | 0.402 | 0.169 | 0.241 | 0.671 | 0.124 | 0.929 |
| rs138389345* | *SLC4A4* | 0.773 | 0.813 | 0.779 | 0.875 | 0.723 | 0.872 | 0.321 | 0.656 | 0.335 | 0.409 |
| rs1453458 | *SLC4A4* | 0.895 | 0.850 | 0.890 | 0.848 | 0.363 | 0.980 | 0.495 | 0.664 | 0.582 | 0.916 |
| rs1453474 | *SLC4A4* | 0.895 | 0.850 | 0.890 | 0.848 | 0.363 | 0.980 | 0.495 | 0.664 | 0.582 | 0.916 |
| rs2291075* | *SLCO1B1* | 0.819 | 0.956 | 0.840 | 0.515 | 0.301 | 0.181 | 0.570 | 0.842 | 0.628 | 0.880 |
| rs4149033* | *SLCO1B1* | 0.894 | 0.743 | 0.463 | 0.802 | 0.926 | 0.403 | 0.750 | 0.613 | 0.221 | 0.570 |
| rs4149056* | *SLCO1B1* | 0.910 | 0.702 | 0.512 | 0.945 | 0.621 | 0.207 | 0.083 | 0.750 | 0.851 | 0.903 |
| rs4149057* | *SLCO1B1* | 0.303 | 0.323 | 0.337 | 0.084 | 0.273 | 0.539 | 0.663 | 0.135 | 0.691 | 0.633 |
| rs11045748* | *SLCO1B7, SLCO1B1* | 0.552 | 0.987 | 0.626 | 0.837 | 0.109 | 0.069 | 0.086 | 0.624 | 0.059 | 0.602 |
| rs11648192 | *SULT1A1* | 0.446 | 0.691 | 0.484 | 0.596 | 0.536 | 0.502 | 0.905 | 0.504 | 0.916 | 0.739 |
| rs2077412 | *SULT1A1* | 0.702 | 0.466 | 0.720 | 0.606 | 0.920 | 0.737 | 0.512 | 0.112 | 0.646 | 0.452 |
| rs28410083 | *SULT1A1* | 0.449 | 0.478 | 0.659 | 0.324 | 0.817 | 0.967 | 0.842 | 0.088 | 0.658 | 0.471 |
| rs3020804 | *SULT1A1* | 0.282 | 0.626 | 0.423 | 0.471 | 0.560 | 0.866 | 0.383 | 0.182 | 0.696 | 0.952 |
| rs3760091 | *SULT1A1* | 0.181 | 0.249 | 0.682 | 0.706 | 0.249 | 0.956 | 0.521 | 0.628 | 0.434 | 0.869 |
| rs9282862* | *SULT1A1* | 0.282 | 0.626 | 0.423 | 0.471 | 0.560 | 0.866 | 0.383 | 0.182 | 0.696 | 0.952 |
| rs4148323* | *UGT1A1* | 0.476 | 0.947 | 0.933 | 0.899 | 0.915 | 0.161 | 0.126 | 0.935 | 0.367 | 0.873 |
| rs4148327* | *UGT1A1,3-10* | 0.886 | 0.415 | 0.902 | 0.319 | 0.755 | 0.244 | 0.216 | 0.509 | 0.859 | 0.973 |
| rs12466997* | *UGT1A3-10* | 0.575 | 0.890 | 0.740 | 0.596 | 0.223 | 0.149 | 0.574 | 0.673 | 0.723 | 0.792 |
| rs2361501* | *UGT1A3-10* | 0.780 | 0.732 | 0.266 | 0.714 | 0.317 | 0.295 | 0.588 | 0.907 | 0.734 | 0.239 |
| rs873478 | *UGT1A3-10* | 0.919 | 0.884 | 0.754 | 0.652 | 0.050 | 0.481 | 0.121 | 0.054 | 0.687 | 0.474 |
| rs869283* | *UGT1A5-10* | 0.993 | 0.620 | 0.322 | 0.659 | 0.892 | 0.692 | 0.825 | 0.423 | 0.400 | 0.193 |
| rs7563561* | *UGT1A7-10* | 0.953 | 0.908 | 0.778 | 0.535 | 0.814 | 0.330 | 0.184 | 0.243 | 0.630 | 0.718 |
| rs7608175* | *UGT1A7-10* | 0.953 | 0.908 | 0.778 | 0.535 | 0.814 | 0.330 | 0.184 | 0.243 | 0.630 | 0.718 |
| rs7586110* | *UGT1A8-10* | 0.695 | 0.981 | 0.919 | 0.605 | 0.850 | 0.723 | 0.305 | 0.365 | 0.802 | 0.843 |
| rs115791839* | *UGT2A3, UGT2B7* | 0.841 | 0.945 | 0.219 | 0.802 | 0.212 | 0.411 | 0.507 | 0.934 | 0.835 | 0.634 |
| rs535517123 | *UGT2A3, UGT2B7* | 0.841 | 0.945 | 0.219 | 0.802 | 0.212 | 0.411 | 0.507 | 0.934 | 0.835 | 0.634 |
| rs12233719* | *UGT2B7* | 0.558 | 0.970 | 0.849 | 0.932 | 0.848 | 0.328 | 0.305 | 0.871 | 0.723 | 0.974 |
| rs28365063* | *UGT2B7* | 0.987 | 0.860 | 0.863 | 0.891 | 0.694 | 0.500 | 0.656 | 0.852 | 0.710 | 0.865 |
| rs4257713* | *UGT2B7* | 0.696 | 0.594 | 0.899 | 0.433 | 0.780 | 0.969 | 0.700 | 0.631 | 0.548 | 0.622 |
| rs5013211* | *UGT2B7* | 0.954 | 0.731 | 0.328 | 0.478 | 0.640 | 0.856 | 0.698 | 0.529 | 0.558 | 0.420 |
| rs7438284* | *UGT2B7* | 0.954 | 0.731 | 0.328 | 0.478 | 0.640 | 0.856 | 0.698 | 0.529 | 0.558 | 0.420 |
| rs7658752* | *UGT2B7* | 0.984 | 0.836 | 0.435 | 0.478 | 0.833 | 0.608 | 0.781 | 0.617 | 0.482 | 0.400 |

*ABCB1*, ATP-binding cassette subfamily B member 1; *RUNDC3B*, RUN Domain Containing 3B; *ABCC2*, ATP Binding Cassette Subfamily C Member 2; *ABCG2*, ATP Binding Cassette Subfamily G Member 2; *CES1*, Carboxylesterase 1; *CYP2A6*, Cytochrome P450 Family 2 Subfamily A Member 6; *CYP2B6*, Cytochrome P450 Family 2 Subfamily B Member 6; *CYP2A13*, Cytochrome P450 Family 2 Subfamily A Member 13; *CYP2C19*, Cytochrome P450 Family 2 Subfamily C Member 19; *CYP2C8*, Cytochrome P450 Family 2 Subfamily C Member 8; *CYP2D6*, Cytochrome P450 Family 2 Subfamily D Member 6; *CYP2AJ2*, Cytochrome P450 Family 2 Subfamily J Member 2; *CYP3A5*, Cytochrome P450 Family 3 Subfamily A Member 5; *ZSCAN25*, Zinc Finger And SCAN Domain Containing 25; *CYP4F2*, Cytochrome P450 Family 4 Subfamily F Member 2; *FRAS1*, Fraser Extracellular Matrix Complex Subunit 1; *SLC22A1*, Solute Carrier Family 22 Member 1; *SLC4A4*, Solute Carrier Family 4 Member 4; *SLCO1B1*, Solute Carrier Organic Anion Transporter Family Member 1B1; *SLCO1B7*, Solute Carrier Organic Anion Transporter Family Member 1B7; *SULT1A1*, Sulfotransferase Family 1A Member 1; *UGT1A1*, UDP Glucuronosyltransferase Family 1 Member A1; *UGT1A3*, UDP Glucuronosyltransferase Family 1 Member A3; *UGT1A4*, UDP Glucuronosyltransferase Family 1 Member A4; *UGT1A5*, UDP Glucuronosyltransferase Family 1 Member A5; *UGT1A6*, UDP Glucuronosyltransferase Family 1 Member A6; *UGT1A7*, UDP Glucuronosyltransferase Family 1 Member A7; *UGT1A8*, UDP Glucuronosyltransferase Family 1 Member A8; *UGT1A9*, UDP Glucuronosyltransferase Family 1 Member A9; *UGT1A10*, UDP Glucuronosyltransferase Family 1 Member A10; *UGT2A3*, UDP Glucuronosyltransferase Family 2 Member A3; *UGT2B7*, UDP Glucuronosyltransferase Family 2 Member B7.

#The SNP was detected and analyzed for variations associated with bleeding in reported pharmacogenomic studies of dabigatran

*The SNP was detected and analyzed for variations associated with pharmacodynamics in reported pharmacogenomic studies of dabigatran

**Table S6. Characteristics of suggestive genes and SNPs associated with PD parameters**

| **Gene** | **ID** | **Location** | **Function** | **Expression** **Tissue*** | **SNP** | **Minor allele** | **MAF(%)#** | | | | | |
| --- | --- | --- | --- | --- | --- | --- | --- | --- | --- | --- | --- | --- |
|  |  |  |  |  |  |  | **All** | **AMR** | **EAS** | **EUR** | **SAS** | **Our results** |
| *SNX7* | 51375 | 1p21.3 | intronic | colon, small intestine, kidney, liver | rs9433747 | G | 14.2 | 4.3 | 10.4 | 4.1 | 4.2 | 8.2 |
| *BRD4* | 23476 | 19p13.12 | exonic | placenta, testis, kidney, liver | rs11669901 | A | 13.2 | 10.1 | 6.2 | 18.8 | 16.4 | 5.9 |
| *FLCN* | 201163 | 17p11.2 | exonic | endometrium, ovary, kidney, liver | rs3744124 | T | 10.0 | 2.0 | 14.9 | 3.3 | 12.5 | 20.6 |
| *UBAP1* | 51271 | 9p13.3 | intronic | bone marrow, testis, kidney, liver | rs1556439 | T | 20.8 | 19.3 | 3.7 | 23.2 | 13.3 | 5.3 |
| *IGLV3-12* | 28802 | 22q11.22 | exonic | no data | rs2073451 | G | 60.9 | 55.6 | 43.1 | 68.9 | 69.8 | 43.8 |
| *LRRC8E* | 80131 | 19p13.2 | exonic | skin, prostate, kidney, liver | rs3745382 | A | 16.3 | 16.7 | 13.4 | 24.5 | 13.5 | 11.5 |
| *PTPLAD1* | 51495 | 15q22.31 | exonic | brain, adrenal, kidney, liver | rs11539008 | A | 11.1 | 14.8 | 7.7 | 17.0 | 8.1 | 7.4 |
| *ZNF230* | 7773 | 19q13.31 | exonic | ovary, thyroid, kidney, liver | rs12753 | A | 22.0 | 29.3 | 25.6 | 17.9 | 10.3 | 24.1 |
| *ANP32A* | 8125 | 15q23 | intronic | lymph node, bone marrow, kidney, liver | rs12904108 | T | 25.5 | 28.7 | 16.4 | 36.2 | 53.3 | 17.4 |
| *SLC25A28* | 81894 | 10q24.2 | intronic | testis, bone marrow, kidney, liver | rs12252561 | C | 25.2 | 25.4 | 19.9 | 24.9 | 31.9 | 19.4 |
| *ABCC2* | 1244 | 10q24.2 | exonic | liver, small intestine, kidney | rs2273697 | A | 18.7 | 15.9 | 9.6 | 20.4 | 27.8 | 8.8 |
|  |  |  |  |  | rs4148395 | A | 19.3 | 16.0 | 9.6 | 20.3 | 27.7 | 8.8 |
| *MYBPC1* | 4604 | 12q23.2 | intronic | prostate, esophagus | rs11110942 | G | 8.6 | 4.2 | 26.0 | 1.9 | 12.0 | 23.5 |
|  |  |  |  |  | rs3751246 | T | 10.3 | 5.0 | 26.1 | 1.9 | 12.0 | 23.8 |
|  |  |  |  |  | rs11110952 | C | 8.7 | 4.2 | 26.0 | 1.9 | 12.5 | 24.1 |
| *CEP170B* | 283638 | 14q32.33 | exonic | colon, brain, kidney, liver | rs60001925 | C | 7.1 | 12.0 | 10.9 | 7.8 | 8.1 | 12.6 |
| *GYPA* | 2993 | 4q31.21 | intronic | bone marrow, placenta, kidney | rs145195209 | A | 9.8 | 10.5 | 10.7 | 4.4 | 4.9 | 9.4 |

*SNX7*, sorting nexin 7; BRD4, bromodomain containing 4; *FLCN*, folliculin; UBAP1, ubiquitin associated protein 1; *IGLV3-12*, immunoglobulin lambda variable 3-12; *LRRC8E*, leucine rich repeat containing 8 VRAC subunit E; *PTPLAD1*, known as HACD3, 3-hydroxyacyl-CoA dehydratase 3; *ZNF230*, zinc finger protein 230; *ANP32A*, acidic nuclear phosphoprotein 32 family member A; *SLC25A28*, solute carrier family 25 member 28; *ABCC2*, ATP binding cassette subfamily C member 2; *MYBPC1*, myosin binding protein C1; *CEP170B*, centrosomal protein 170B; *GAPA*, glycophorin A (MNS blood group); MAF, minor allele frequency; AMR, American; EAS, East Asian; EUR, European; SAS, South Asian.

*From the HPA RNA-seq normal tissue data. The two most distributed tissues are listed. If expressed in kidney or liver, list it.

#From the 1000 Genomes Project Phase 3 populations data.

**References**

[1] Gouin-Thibault I, Delavenne X, Blanchard A, Siguret V, Salem JE, Narjoz C, *et al*. Interindividual variability in dabigatran and rivaroxaban exposure: contribution of ABCB1 genetic polymorphisms and interaction with clarithromycin. *J Thromb Haemost*. 2017; 15: 273-83.

[2] Zubiaur P, Saiz-Rodríguez M, Ochoa D, Navares-Gómez M, Mejía G, Román M, et al. Effect of Sex, Use of Pantoprazole and Polymorphisms in SLC22A1, ABCB1, CES1, CYP3A5 and CYP2D6 on the Pharmacokinetics and Safety of Dabigatran. *Adv Ther*. 2020, 37 (8): 3537–3550.

[3] Liu Y, Yang C, Qi W, Pei Z, Xue W, Zhu H, et al. The Impact of ABCB1 and CES1 Polymorphisms on Dabigatran Pharmacokinetics in Healthy Chinese Subjects. *Pharmgenomics Pers Med*. 2021, 14:477-485.

[4] Xie QF, Li Y, Liu ZY, Mu GY, Zhang HX, Zhou S, et al. SLC4A4, FRAS1, and SULT1A1 genetic variations associated with dabigatran metabolism in a healthy Chinese population. *Front. Genet.* 2022, 13:873031.

[5] Paré G, Eriksson N, Lehr T, Connolly S, Eikelboom J, Ezekowitz MD, et al. Genetic determinants of dabigatran plasma levels and their relation to bleeding. *Circulation*. 2013, 127(13):1404-1412.

[6] Chin PK, Wright DF, Zhang M, Wallace MC, Roberts RL, Patterson DM, et al. Correlation between trough plasma dabigatran concentrations and estimates of glomerular filtration rate based on creatinine and cystatin C. *Drugs R D*. 2014;14(2): 113–123.

[7] Tomita H, Araki T, Kadokami T, Yamada S, Nakamura R, Imamura Y, et al. Factors influencing trough and 90-minute plasma dabigatran etexilate concentrations among patients with non-valvular atrial fibrillation. *Thromb Res*. 2016, 145:100-106.

[8] Dimatteo C, D'Andrea G, Vecchione G, Paoletti O, Cappucci F, Tiscia GL, *et al*. Pharmacogenetics of dabigatran etexilate interindividual variability. *Thromb Res*. 2016; 144: 1-5.

[9] Sychev D, Skripka A, Ryzhikova K, Bochkov P, Shevchenko R, Krupenin P, et al. Effect of CES1 and ABCB1 genotypes on the pharmacokinetics and clinical outcomes of dabigatran etexilate in patients with atrial fibrillation and chronic kidney disease. *Drug Metab Pers Ther*. 2020;35(1):/j/dmdi.2020.35.issue-1/dmpt-2019-0029/dmpt-2019-0029.xml.

[10] Roşian AN, Iancu M, Trifa AP, Roşian ŞH, Mada C, Gocan CP, et al. An Exploratory Association Analysis of ABCB1 rs1045642 and rs4148738 with Non-Major Bleeding Risk in Atrial Fibrillation Patients Treated with Dabigatran or Apixaban. *J Pers Med*. 2020;10(3): 133.

[11] Ji Q, Zhang C, Xu Q, Wang Z, Li X, Lv Q. The impact of ABCB1 and CES1 polymorphisms on dabigatran pharmacokinetics and pharmacodynamics in patients with atrial fibrillation. *Br J Clin Pharmacol*. 2021, 87(5):2247-2255.

[12] Lähteenmäki J, Vuorinen AL, Pajula J, Harno K, Lehto M, Niemi M, et al. Pharmacogenetics of Bleeding and Thromboembolic Events in Direct Oral Anticoagulant Users. *Clin Pharmacol The*r. 2021, 110(3):768-776.
