# Supplementary material for: Genetic variations in relation to bleeding and pharmacodynamics of dabigatran in Chinese patients with nonvalvular atrial fibrillation: A nationwide multicentre prospective cohort study
Source: Clin Transl Med. 2022 Dec 1;12(12):e1104. doi: 10.1002/ctm2.1104 (PMC9714378; doi:10.1002/ctm2.1104)

**Supplementary 2. Figures**

Figure S1: Manhattan plots of association with pharmacodynamic parameters.

1. FIIa

**
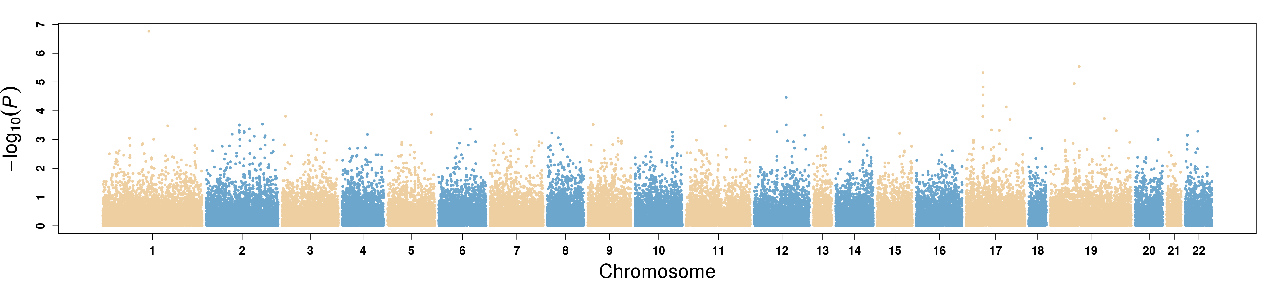
**

2. FAPTT


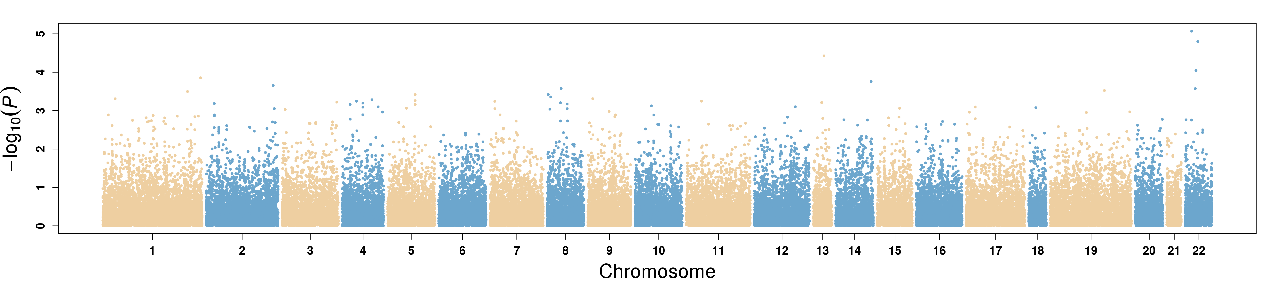


3. FPT


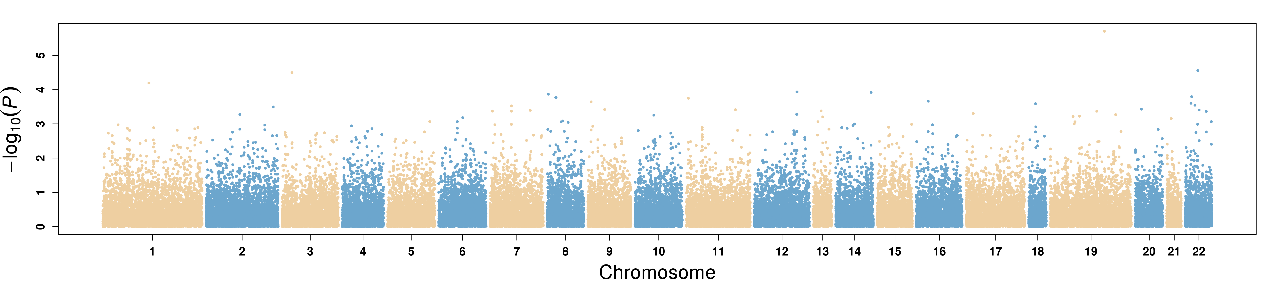


4. GIIa


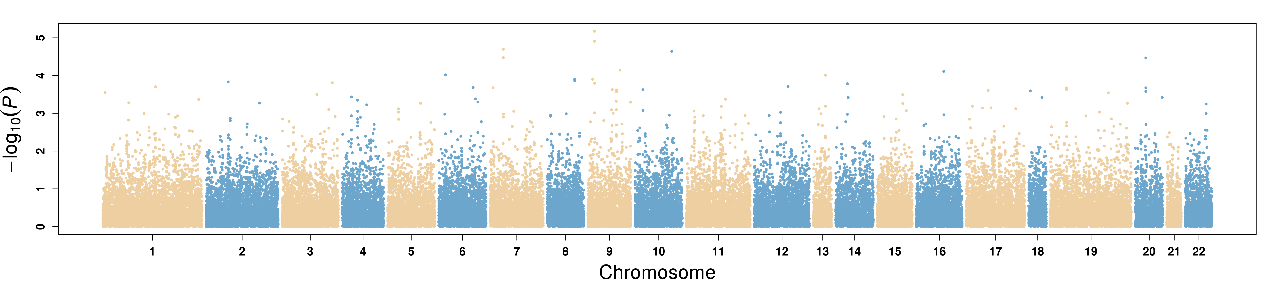


5. GAPTT


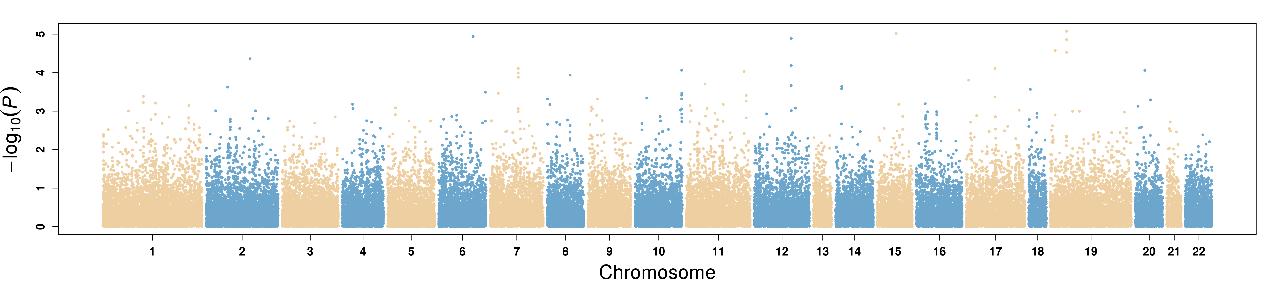


6. GPT


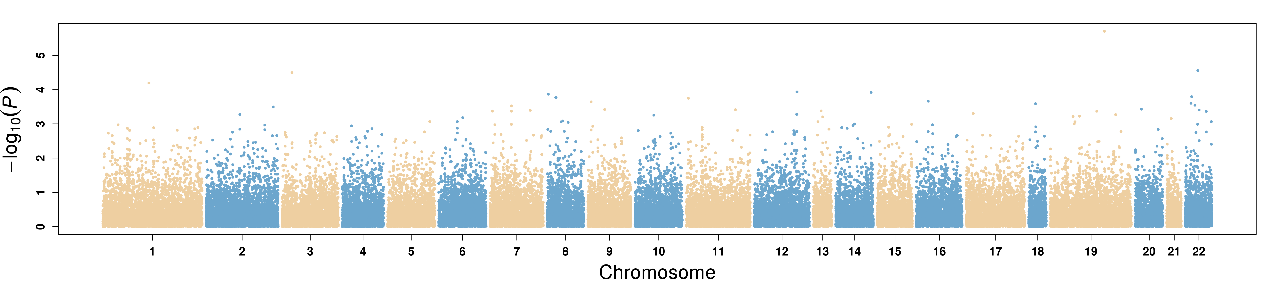


Figure S2: Quantile–quantile plots of association with pharmacodynamic parameters.

1. FIIa


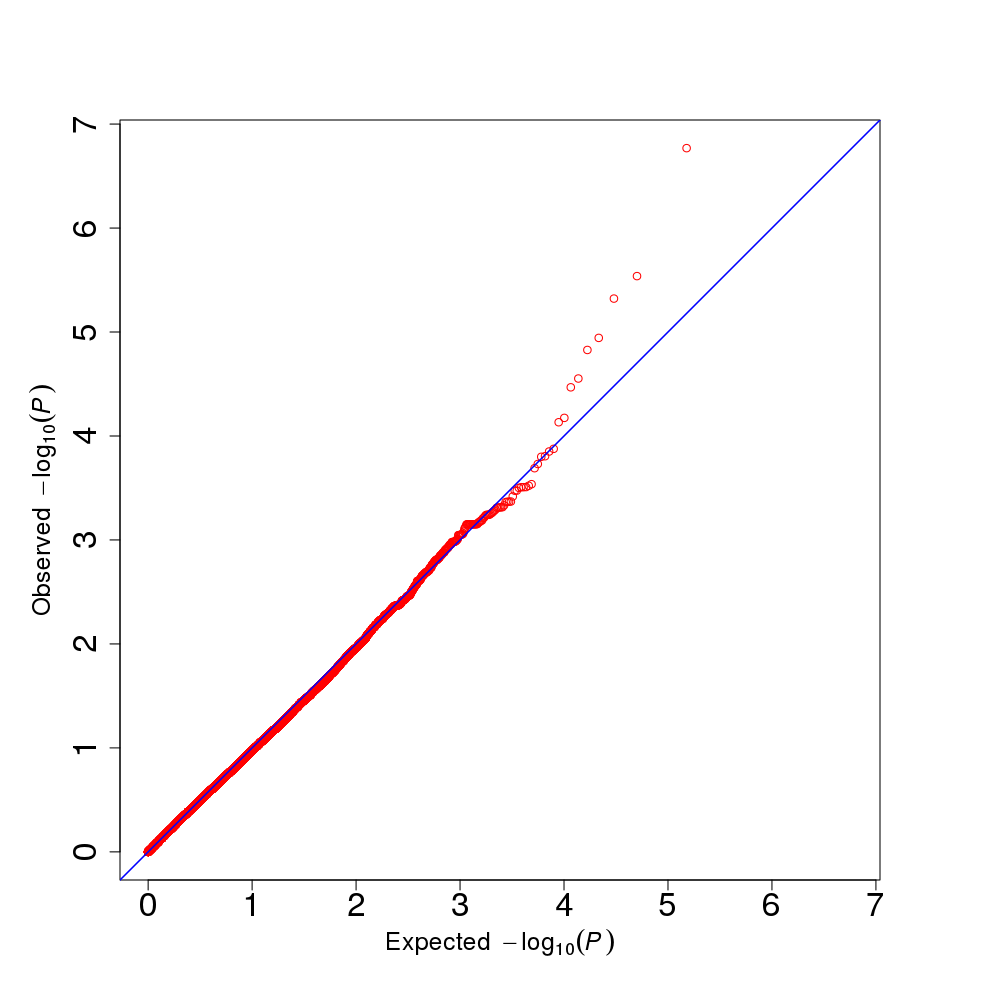


2. FAPTT


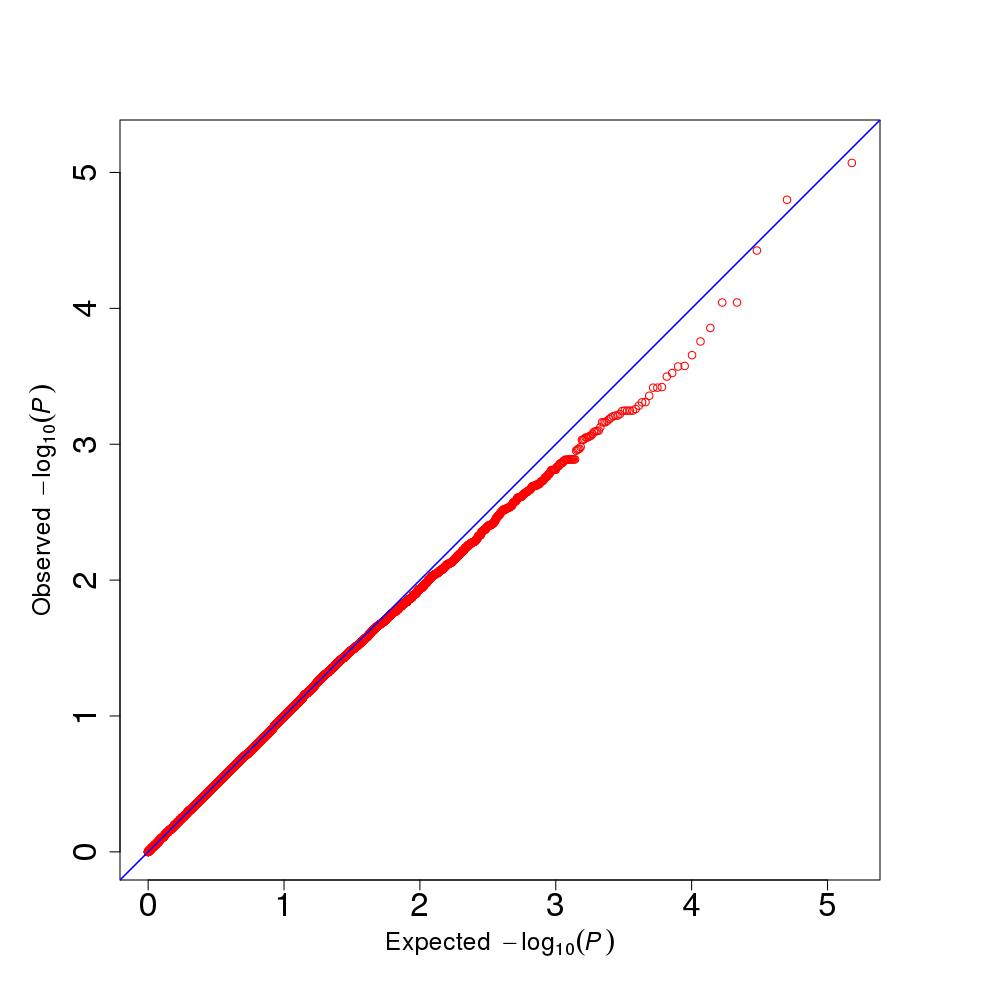


3. FPT


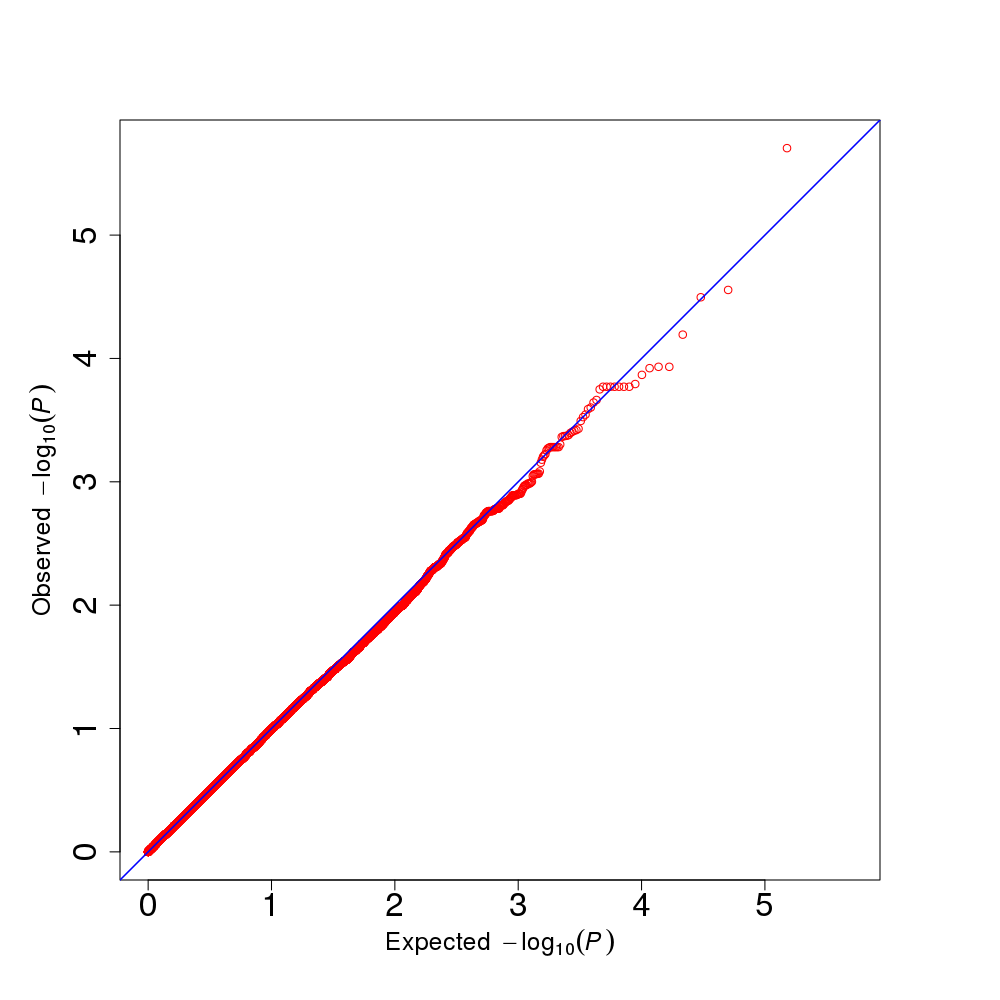


4. GIIa


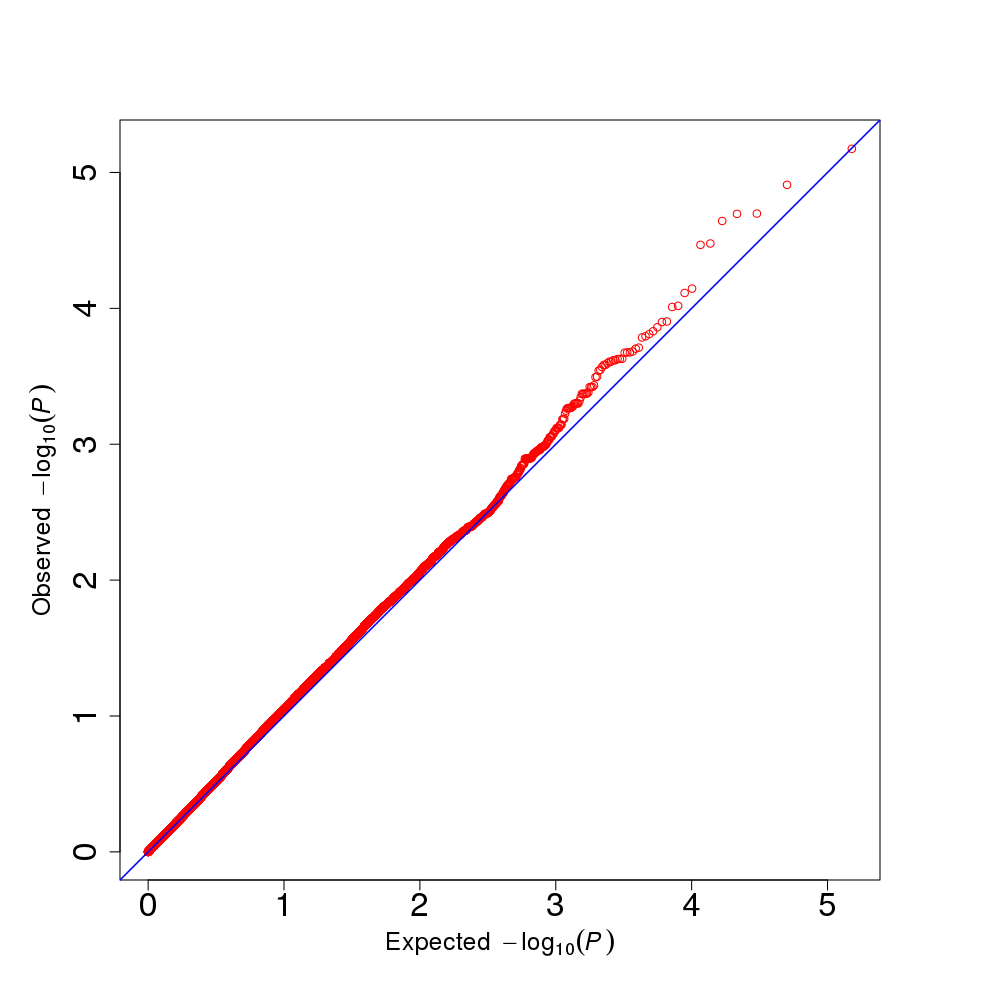


5. GAPTT


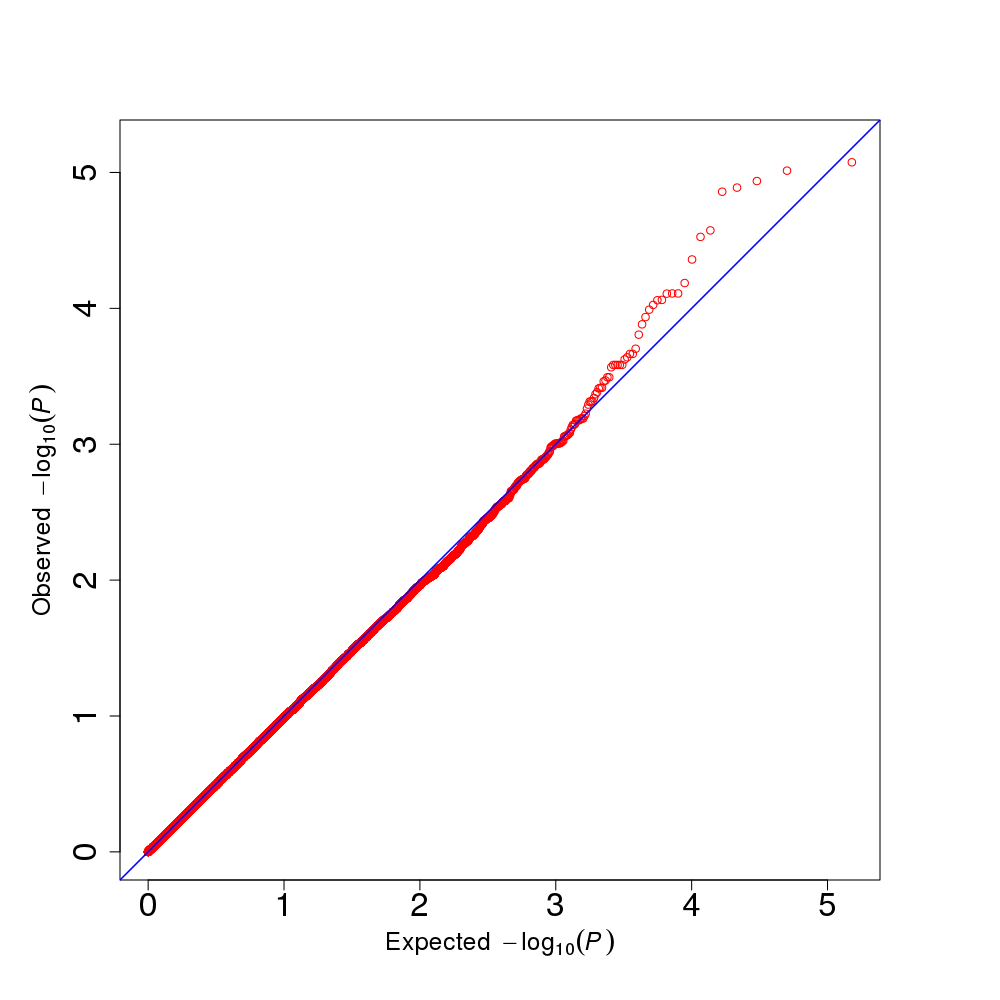


6. GPT


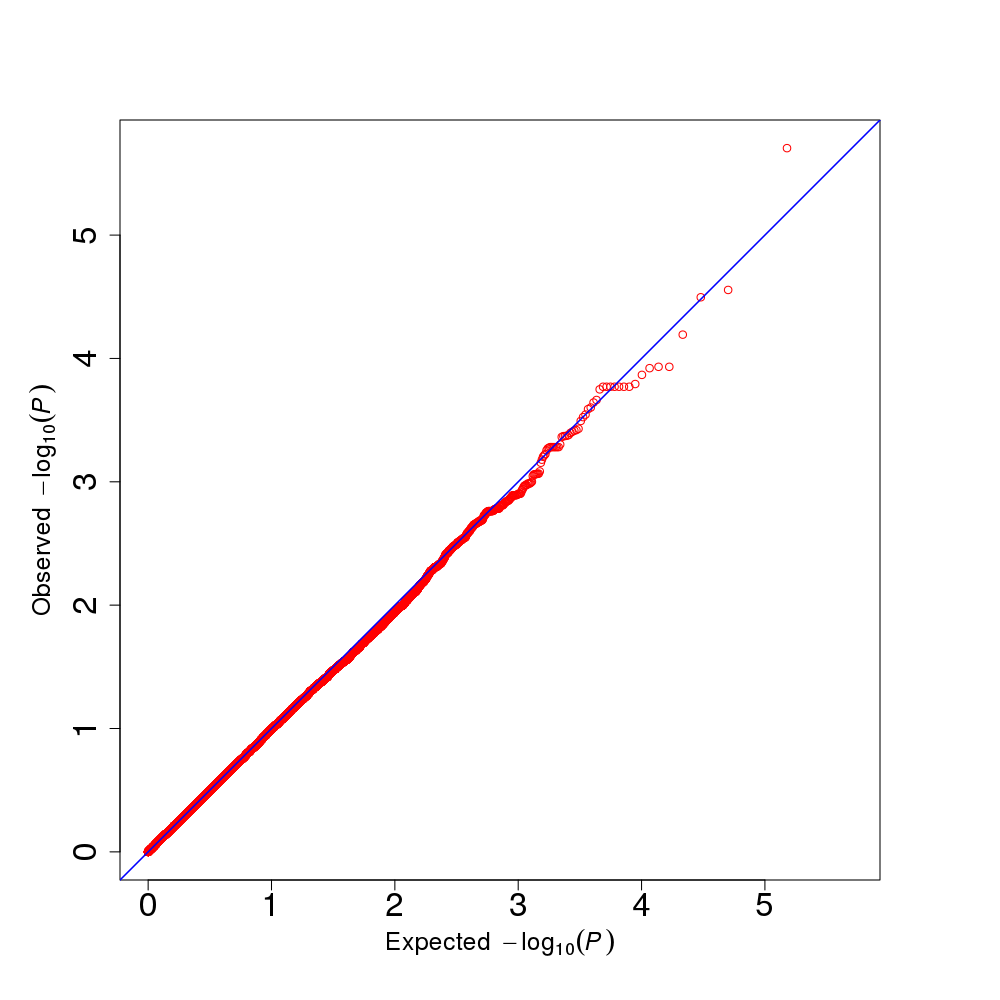

Supplement: Supplementary file 2 — Figure S1 Manhattan plots of association with pharmacodynamic parameters Figure S2 Quantile–quantile plots of association with pharmacodynamic parameters [file CTM2-12-e1104-s001.docx]
